# GSK3α functions as a stemness checkpoint across multiple stem cell states

**DOI:** 10.1038/s41422-026-01245-5

**Published:** 2026-04-09

**Authors:** Duo Wang, Xiukun Wang, Safia Malki, Yanpui Chan, Brian Bennett, Joshua Feng, Jiaqi Tang, Xi Chen, Daniel McKim, Chao Zhang, Litao Tao, Jie Xu, Y. Eugene Chen, Guang Hu, Qi-Long Ying

**Affiliations:** 1 Eli and Edythe Broad Center for Regenerative Medicine and Stem Cell Research at USC, Department of Stem Cell Biology and Regenerative Medicine, Keck School of Medicine, University of Southern California, Los Angeles, CA USA; 2 Center for Advanced Models Translational Sciences and Therapeutics, University of Michigan Medical School, Ann Arbor, MI USA; 3 Epigenetics and RNA Biology Laboratory, National Institute of Environmental Health Sciences, Durham, NC USA; 4 Loker Hydrocarbon Research Institute & Department of Chemistry, University of Southern California, Los Angeles, CA USA; 5 Department of Biomedical Sciences, School of Medicine, Creighton University, Omaha, NE USA

**Keywords:** Stem cells, Pluripotency, Self-renewal

Dear Editor,

Pluripotent stem cells (PSCs) generate derivatives of all three germ layers; however, robust contribution to chimeric embryos is largely restricted to early pluripotent states. Mouse embryonic stem cells (mESCs), derived from the inner cell mass, readily contribute to chimeras, whereas post-implantation mouse epiblast-derived stem cells (mEpiSCs) lack this developmental competence.^1,2^ mESC self-renewal is maintained under the 2i condition (PD0325901 + CHIR99021), which inhibits FGF/MEK signaling and activates WNT signaling, whereas mEpiSCs require AFX (activin A, bFGF, and XAV939).^1,3^ mESCs readily transition to an mEpiSC-like state under AFX conditions, but the reverse transition rarely occurs, highlighting fundamental differences in the regulatory logic underlying ESC and EpiSC pluripotency.^1,2^ Nonetheless, the molecular mechanisms that stabilize self-renewal across distinct pluripotent states remain incompletely understood.

To test the hypothesis that PSCs with distinct developmental identities may nevertheless share common intrinsic mechanisms supporting self-renewal, we performed a small-molecule screen to identify factors capable of sustaining both mESCs and mEpiSCs. These two PSC types rely on largely opposing signaling pathways for self-renewal, making them a stringent system for uncovering shared regulatory principles. We screened a focused library of 164 compounds targeting pathways involved in cell proliferation and stemness and identified multiple candidates that enhanced mESC colony formation. Five top-ranking compounds (BRD0705, acetylcysteine, TG100-115, doramapimod, and IWR1) were selected for validation (Supplementary information, Fig. S1a). Among these, BRD0705, a selective GSK3α inhibitor,^4^ uniquely sustained mESC self-renewal during long-term passaging in the absence of 2i/LIF (2iL), as confirmed by alkaline phosphatase staining and OCT4-GiP reporter ESCs^5^ (Supplementary information, Fig. S1b–g). BRD0705 alone was sufficient to maintain 129P2/Ola and B6D2F1 mESCs in an undifferentiated state during long-term culture, with preserved expression of pluripotency markers and full reversibility upon return to 2iL (Fig. 1a; Supplementary information, Fig. S1h, i). Notably, BRD0705 replaced the pan-GSK3 inhibitor CHIR99021 (CHIR) in N2B27 + PD0325901 (PD03), supporting the derivation and long-term expansion of chimera-competent mESCs (Supplementary information, Fig. S2a–d). A parallel screen in mEpiSCs also identified BRD0705 among the top candidates (Fig. 1b; Supplementary information, Fig. S3a). These findings prompted us to investigate whether GSK3α functions as a common regulatory node that constrains differentiation across PSC states.

**Fig. 1:**
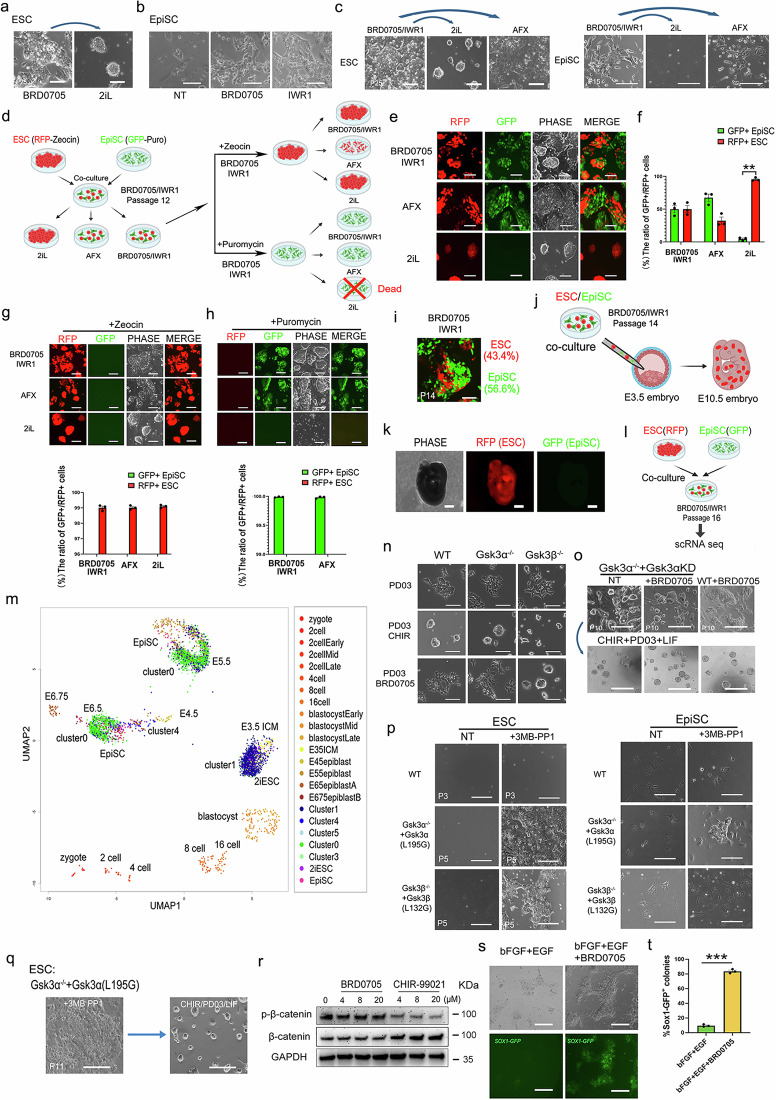
Selective GSK3α inhibition promotes self-renewal across different stem cell states. **a** Left: phase-contrast image of mESCs at passage 25 (P25) cultured in DMEM supplemented with 10% FBS + BRD0705. Right: mESCs cultured in 2iL for three passages after P25 in BRD0705. Scale bars, 100 μm. **b** Representative images of mEpiSCs treated with BRD0705 or IWR1 from the compound library on day 3. Scale bars, 100 μm. NT, non-treated. **c** Left: images of mESCs after P25 in BI, followed by 3 passages in 2iL or AFX. Scale bars, 100 μm. Right: mEpiSCs after P15 in BI, followed by 3 passages in 2iL or AFX. Scale bars, 200 μm. **d** Schematic of RFP^+^ mESCs and GFP^+^ mEpiSCs co-cultured in BI for 12 passages, followed by culture-condition switching and antibiotic selection with zeocin or puromycin to isolate each cell type. **e** Bright-field and fluorescent images of RFP^+^ mESCs and GFP^+^ mEpiSCs from P12 BI co-culture after transition to BI, AFX, or 2iL. Scale bars, 100 μm. **f** Bar graph showing the ratio of mESCs (RFP-zeocin) and mEpiSCs (GFP-puromycin) in **e** under BI, AFX, and 2iL conditions. **g** Bright-field and fluorescent images of RFP^+^ mESCs and GFP^+^ mEpiSCs after zeocin selection and 3 passages in BI, AFX, or 2iL. Scale bars, 100 μm. The bar graph shows GFP^+^ and RFP^+^ cell proportions. **h** Bright-field and fluorescent images of RFP^+^ mESCs and GFP^+^ mEpiSCs after puromycin selection and 2 passages in BI, AFX, or 2iL. Scale bars, 100 μm. The bar graph shows GFP^+^ and RFP^+^ cell proportions. **i** Representative morphology of P14 co-cultured mESCs (RFP^+^) and mEpiSCs (GFP^+^) under BI conditions used for injection into WT E3.5 blastocysts, together with the respective proportions of these two cell types. Scale bar, 100 μm. **j** Schematic of chimeric embryo generation by injection of P14 BI co-cultured RFP^+^ mESCs/GFP^+^ mEpiSCs into WT E3.5 embryos, showing their contribution to E10.5 development. **k** Images of E10.5 embryos derived from E3.5 blastocysts injected with P14 BI co-cultured RFP^+^ mESCs/GFP^+^ mEpiSCs, showing cell distribution and integration. Scale bar, 500 μm. **l** Schematic illustration of the experimental design. mESCs (RFP^+^) and mEpiSCs (GFP^+^) were co-cultured under BI conditions for 16 passages, followed by scRNA-seq analysis. **m** UMAP clustering of single-cell transcriptomes reveals distinct co-culture cell populations projected onto a UMAP plot of embryonic developmental stages. Clusters 1–5 correspond to those shown in Supplementary information, Fig. S5b. scRNA-seq reference data for embryonic developmental stages were obtained from publicly available datasets (GSE45719 and GSE100597), and reference data for 2i-cultured mESCs and mEpiSCs were obtained from GSE74155. **n** Morphology of WT, *Gsk3α*^*–/–*^, and *Gsk3β*^*–/–*^ mESCs cultured in N2B27 for 4 days with PD03, PD03 + CHIR, or PD03 + BRD0705. Scale bars, 200 µm. **o** Top row: *Gsk3α*^*–/–*^ *+ Gsk3α-KD* mESCs under NT and BRD0705 conditions and WT mESCs treated with BRD0705 after 10 passages. Bottom row: morphology of the corresponding P10 cells after transition to 2iL conditions for 3 passages. Scale bars, 100 µm. **p** Morphology of WT, *Gsk3α*^*–/–*^ *+ Gsk3α-L195G*, and *Gsk3β*^*–/–*^ *+ Gsk3β-L132G* ESCs and EpiSCs under different treatments. Representative images show cells cultured in DMEM/FBS medium for 3 passages under NT or + 3MB-PP1 conditions. Scale bars, 200 μm. **q** Morphology of *Gsk3α*^*–/–*^ *+ Gsk3α-L195G* mESCs under different culture conditions. Representative images of cells at P11 cultured in + 3MB-PP1 (left), followed by a switch to 2iL (right). Scale bars, 200 μm. **r** Western blot analysis of phosphorylated β-catenin (p-β-catenin), total β-catenin, and GAPDH in response to different concentrations of BRD0705 and CHIR. **s** Representative images illustrating the effect of BRD0705 treatment on neural stem cell expansion. Scale bars, 200 μm. **t** Quantification of **s**. The bar graph shows the percentage of SOX1-GFP^+^ colonies in neural stem cells under control and BRD0705 treatment conditions (*P < 0.05). Data are presented as mean ± SEM. Illustrations in **d**, **j** and **l** are created with Biorender.

Although BRD0705 alone supported only short-term mEpiSC self-renewal (Supplementary information, Fig. S3b), a secondary screen identified the tankyrase inhibitor IWR1 as a cooperating factor (Fig. 1b; Supplementary information, Fig. S1a–c). Combined treatment with BRD0705 + IWR1 (BI) enabled long-term expansion of both mESCs and mEpiSCs (Fig. 1c). mESCs cultured under BI retained developmental plasticity, adapting to either 2iL or AFX conditions and contributing to germ cells in embryonic day 15.5 (E15.5) gonads (Supplementary information, Fig. S4h–k). In contrast, BI-maintained mEpiSCs adapted to AFX but failed to grow in 2iL, consistent with the unidirectional ESC-to-EpiSC transition^1,2^ (Fig. 1c).

mESCs and mEpiSCs cultured under BI expressed the core pluripotency markers NANOG and OCT4, lacked lineage markers (*Foxa2* and *Gata4*), and retained tri-lineage differentiation potential (Supplementary information, Fig. S4a–d). Consistent with their respective identities, mESCs exhibited high *Rex1* and low *Fgf5/Otx2* expression, whereas mEpiSCs displayed the opposite pattern characteristic of the primed state (Supplementary information, Fig. S4a, b). Functionally, only BI-expanded mESCs contributed to chimeras and germline cells, whereas mEpiSCs failed to do so (Supplementary information, Fig. S1e–l and Video S1).

Remarkably, BI maintained two distinct pluripotent states within a single culture condition. RFP-labeled mESCs (zeocin-resistant) and GFP-labeled mEpiSCs (puromycin-resistant) were co-cultured at a 1:1 ratio under BI for more than one month (Fig. 1d–f; Supplementary information, Fig. S4m). Co-cultured cells were subsequently subjected to antibiotic selection. Zeocin-selected RFP^+^ mESCs proliferated robustly under BI, AFX, and 2iL conditions (Fig. 1g), confirming the retention of ESC identity, whereas puromycin-selected GFP^+^ mEpiSCs survived only in BI and AFX and failed to grow in 2iL (Fig. 1h), consistent with maintenance of the primed state. After 14 passages (>1 month), the mixed BI-cultured population (43.4% ESCs and 56.6% EpiSCs) was injected into mouse blastocysts (Fig. 1i, j). RFP^+^ mESCs contributed to E10.5 chimeras (55.6%), whereas GFP^+^ mEpiSC-derived cells were completely absent, reflecting their limited developmental competence compared with the high chimeric potential of RFP^+^ mESCs (Fig. 1k; Supplementary information, Fig. S4n, o).

Single-cell RNA sequencing (scRNA-seq) of co-cultured RFP^+^ mESCs and GFP^+^ mEpiSCs after 16 passages under BI conditions, followed by uniform manifold approximation and projection (UMAP) analysis, revealed three distinct clusters corresponding to RFP^+^ mESCs, GFP^+^ mEpiSCs, and GFP^–^/RFP^–^/vimentin^+^ feeder cells (Supplementary information, Fig. S5a, b). Both mESCs and mEpiSCs expressed *Nanog*. Naïve pluripotency markers (*Esrrb*, *Zfp42*, *Nr0b1*, *Tfcp2l1*, *Tbx3*, *Tcl1*, *and Prdm14*) were enriched in the RFP^+^ mESC cluster, whereas primed markers (*Fgf5* and *Pitx2*) were largely restricted to the GFP^+^ mEpiSC cluster (Supplementary information, Fig. S5c). The GFP^+^ and RFP^+^ populations were further subdivided, with clusters 0 and 1 representing the major groups (Supplementary information, Fig. S5d). UMAP projection onto embryonic reference datasets showed that RFP^+^ mESCs aligned with E3.5 embryos, whereas GFP^+^ mEpiSCs aligned with the E5.5–E6.5 epiblast (Fig. 1m). Hierarchical clustering further confirmed transcriptomic similarity to mESCs cultured in 2i and mEpiSCs cultured in activin A/bFGF (Fig. 1m; Supplementary information, Fig. S5e, f), demonstrating that the two populations retained their developmental identities after extended co-culture. Consistent with these results, epigenetic profiling of H3K4me3, H3K27ac, and H3K27me3 by CUT&Tag in BI co-cultured cells revealed distinct, cell type-specific chromatin states closely resembling those of ESCs and EpiSCs (Supplementary information, Fig. S5g, h). Bivalent gene profiles from co-cultured RFP^+^ (putative naïve) and GFP^+^ (putative primed) cells under BI conditions showed substantial overlap with previously reported bivalent gene datasets (GSE15626137), with concordance rates of 82.57% (naïve bivalent genes) and 56.86% (primed bivalent genes), respectively (Supplementary information, Fig. S5i). These findings are consistent with previously described pluripotent chromatin states in ESCs and EpiSCs. Together, these data demonstrate that BI maintains mESC and mEpiSC populations with distinct and stable transcriptional and epigenetic identities during prolonged co-culture.

We next investigated whether BRD0705 promotes mESC and mEpiSC self-renewal through selective inhibition of GSK3α and whether this effect involves β-catenin. In *Gsk3α/β* double-knockout mESCs cultured in N2B27, self-renewal was sustained by PD03 alone, and the addition of either BRD0705 or CHIR provided no further benefit (Supplementary information, Fig. S6a–d). *Gsk3α*^*–/–*^ and *Gsk3β*^*–/–*^ mESCs were then used to determine whether BRD0705 specifically inhibits GSK3α. When combined with PD03, CHIR maintained self-renewal in wild-type (WT), *Gsk3α*^*–/–*^, and *Gsk3β*^*–/–*^ mESCs (Fig. 1n). In contrast, under the same PD03 condition, BRD0705 was sufficient to sustain self-renewal in *Gsk3β*^*–/–*^ mESCs but not in *Gsk3α*^*–/–*^ mESCs (Fig. 1n; Supplementary information, Fig. S6e, f), indicating that BRD0705 acts through selective inhibition of GSK3α rather than GSK3β. To further validate these findings, we used mESCs in which endogenous GSK3α was replaced with a kinase-dead (KD) mutant (*GSK3α-K148R*).^6^ This strategy selectively disrupts kinase activity that is otherwise lost in *Gsk3*^*–/–*^ mESCs. Under DMEM + 10% FBS conditions without additional inhibitors, *Gsk3α*^*–/–*^ + *Gsk3α-KD* mESCs remained undifferentiated, whereas WT mESCs differentiated or died by passage 3 (Supplementary information, Fig. S6g). Moreover, both WT mESCs cultured with BRD0705 and *Gsk3α*^*–/–*^ + *Gsk3α-KD* mESCs cultured without BRD0705 remained undifferentiated after ten passages and upon transfer to 2iL (Fig. 1o), suggesting that selective inhibition of GSK3α kinase activity is sufficient to maintain mESC self-renewal.

A complementary chemical-genetic approach using GSK3α-L195G and GSK3β-L132G mutants^6^ showed that selective GSK3α inhibition by 3MB-PP1 sustained mESC and mEpiSC self-renewal, whereas selective GSK3β inhibition induced mEpiSC differentiation (Fig. 1p, q). Biochemical analyses further revealed that, unlike CHIR, BRD0705 functioned in β-catenin-deficient cells, did not alter β-catenin phosphorylation, and retained its ability to promote self-renewal in the absence of β-catenin (Fig. 1r; Supplementary information, Fig. S7a–f). Consistent with these observations, luciferase assays showed that CHIR robustly activated the TopFlash reporter in WT mESCs but not in *β-catenin*^*–/–*^ mESCs, whereas BRD0705 failed to activate TopFlash in either genotype (Supplementary information, Fig. S7b), aligned with previous findings in acute myeloid leukemia cells.^4^ Nonetheless, BRD0705 effectively promoted self-renewal in both WT and *β-catenin*^*–/–*^ mESCs (Supplementary information, Fig. S7c–e). Thus, unlike CHIR, which acts through the canonical WNT/β-catenin pathway, BRD0705 sustains mESC self-renewal via β-catenin-independent selective inhibition of GSK3α, revealing a noncanonical mechanism of pluripotency regulation.

Finally, to examine whether this GSK3α-dependent regulatory mechanism extends beyond ESCs and EpiSCs, we cultured additional stem cell types under BI conditions. Mouse formative stem cells maintained in A_lo_XR conditions^1^ retained their formative identity after long-term culture in BI and could revert to A_lo_XR with comparable gene expression profiles (Supplementary information, Fig. S8a, b). In addition, SOX1^+^ mouse neural stem cells cultured with BRD0705 under bFGF/EGF conditions maintained SOX1 expression for longer periods than controls (Fig. 1s, t). Together, these results suggest that GSK3α functions as a central regulator of self-renewal across diverse stem cell types, including ESCs, EpiSCs, formative stem cells, and neural stem cells.

In this study, we identified GSK3α as a regulator of stem cell self-renewal whose effects are strongly dependent on cellular context and culture conditions. Selective inhibition of GSK3α stabilizes stem cells within their existing identities under defined signaling environments, limiting both premature differentiation and inappropriate transitions between pluripotent states. This effect is observed in ESCs, EpiSCs, formative PSCs, and neural stem cells, suggesting a conserved mechanism operating across distinct developmental contexts. However, in each case, sustained self-renewal requires cooperation with specific extrinsic cues. Our findings therefore support a context-dependent model in which GSK3α inhibition enhances stem cell stability but does not autonomously impose or maintain pluripotency.

Our data demonstrate that BRD0705 promotes stem cell self-renewal through selective inhibition of GSK3α via a β-catenin-independent mechanism. In contrast to CHIR, which inhibits both GSK3 isoforms and activates canonical WNT signaling, BRD0705 maintains self-renewal without stabilizing β-catenin, thereby avoiding the differentiation-inducing effects observed in mEpiSCs and human PSCs.^6,7^ This distinction may explain why BRD0705 supports self-renewal under conditions in which canonical WNT signaling is insufficient or detrimental. Consistent with this interpretation, a separate study demonstrated that ESCs or iPSCs from rat, rabbit, bovine, and human can be derived and maintained using a culture system in which selective GSK3α inhibition constitutes a core component, despite the requirement for additional species-specific factors.^8^ Together, these findings support the view that GSK3α inhibition represents a broadly conserved regulatory axis for stabilizing pluripotency across developmental states and species.

Mechanistically, our results implicate the PI3K/AKT/mTOR axis as a key downstream effector of GSK3α inhibition (Supplementary information, Figs. S9, S10). BRD0705 activates p70S6K phosphorylation, and pharmacological inhibition of PI3K abolishes its self-renewal-promoting effects (Supplementary information, Fig. S10). These findings are consistent with previous studies showing that sustained AKT/mTOR activity supports long-term pluripotency in both rodent and primate stem cells.^9–11^ Our previous work demonstrated that selective inhibition of GSK3β promotes mESC self-renewal under N2B27-only or N2B27/PD03 conditions through activation of the canonical β-catenin-dependent WNT pathway.^6^ However, this effect is not universal, as GSK3β inhibition induces differentiation of mEpiSCs and fails to support self-renewal of non-rodent ESCs. In the same study, GSK3α inhibition alone was insufficient to maintain mESC self-renewal under feeder- and serum-free conditions. Consistent with this finding, we show here that GSK3α inhibition is insufficient to sustain mESC self-renewal in N2B27 alone but effectively maintains self-renewal in the presence of serum or feeder cells (Supplementary information, Fig. S11), indicating cooperation with extrinsic signals. This requirement parallels LIF signaling, which also depends on serum or BMP support to maintain mESC self-renewal.^12^ Notably, unlike GSK3β inhibition, GSK3α inhibition maintains self-renewal across multiple pluripotent states, including ESCs, EpiSCs, and formative PSCs, and also supports neural stem cells, highlighting its broader role in stemness regulation.

The BI condition uniquely enables long-term coexistence of ESCs and EpiSCs without interconversion, even during prolonged co-culture. This stability likely reflects complementary signaling effects: GSK3α inhibition promotes intrinsic self-renewal, whereas IWR1 suppresses canonical WNT signaling to preserve the primed identity. Together, these effects create a balanced microenvironment that permits the parallel maintenance of distinct pluripotent states.

We therefore propose that GSK3α functions as a stemness checkpoint. Its inhibition restrains differentiation and stabilizes stem cell identity across multiple stem cell states. Rather than specifying a particular pluripotent identity, this checkpoint acts permissively, buffering stem cells against differentiation cues and inappropriate state transitions. This mechanism is conceptually distinct from lineage-defining transcriptional networks and instead represents a modulatory layer that safeguards stemness across diverse signaling contexts.

Our findings raise the possibility that additional kinases or signaling nodes may serve similar stabilizing roles in other stem cell types. Although this broader principle remains to be tested, systematic identification of such checkpoint-like mechanisms could reveal a general strategy by which stem cells preserve identity despite fluctuating extrinsic signals and may inform the development of more robust, potentially species-independent culture conditions.

## Supplementary information

Supplementary information

Supplementary information, Table S1

Supplementary information, Table S2

Supplementary information, Table S3

Supplementary information, Video S1

## Data Availability

All study data are included in the article and/or supplementary information. The CUT&Tag data have been deposited in the Gene Expression Omnibus (GEO) under accession number GSE295265. The scRNA-seq data have been deposited in GEO under accession number GSE295264. The bulk RNA-seq data have been deposited in GEO under accession numbers GSE295263 and GSE304689.

## References

[1] Kinoshita, M. et al. *Cell Stem Cell* **28**, 453–471.e8 (2021). 33271069 10.1016/j.stem.2020.11.005 PMC7939546

[2] Nichols, J. & Smith, A. *Cell Stem Cell* **4**, 487–492 (2009). 19497275 10.1016/j.stem.2009.05.015

[3] Ying, Q. L. et al. *Nature* **453**, 519–523 (2008). 18497825 10.1038/nature06968 PMC5328678

[4] Wagner, F. F. et al. *Sci. Transl. Med.* **10**, eaam8460 (2018). 29515000 10.1126/scitranslmed.aam8460 PMC6553635

[5] Ying, Q. L., Nichols, J., Evans, E. P. & Smith, A. G. *Nature* **416**, 545–548 (2002). 11932748 10.1038/nature729

[6] Chen, X. et al. *Dev. Cell* **43**, 563–576.e4 (2017). 29207259 10.1016/j.devcel.2017.11.007 PMC5851779

[7] Kim, H. et al. *Nat. Commun.* **4**, 2403 (2013). 23985566 10.1038/ncomms3403 PMC4183150

[8] Wang, D. et al. *bioRxiv*10.1101/2025.05.20.654948 (2025).

[9] Watanabe, S. et al. *Oncogene* **25**, 2697–2707 (2006). 16407845 10.1038/sj.onc.1209307

[10] Wamaitha, S. E. et al. *Nat. Commun*. **11**, 764 (2020). 10.1038/s41467-020-14629-x PMC7005693 32034154

[11] Zhou, J. et al. *Proc. Natl. Acad. Sci. USA.* **106**, 7840–7845 (2009). 19416884 10.1073/pnas.0901854106 PMC2683106

[12] Ying, Q. L., Nichols, J., Chambers, I. & Smith, A. *Cell* **115**, 281–292 (2003). 14636556 10.1016/s0092-8674(03)00847-x

