# Supplementary material for: GSK3α functions as a stemness checkpoint across multiple stem cell states
Source: Cell Res. 2026 Apr 9;36(8):633–6. doi: 10.1038/s41422-026-01245-5 (PMC13361078; doi:10.1038/s41422-026-01245-5)
Supplement: Supplementary file 1 — Supplementary information [file 41422_2026_1245_MOESM1_ESM.pdf]

Supplementary information

GSK3α functions as a stemness checkpoint across multiple stem cell states

Duo Wang[1,2,6✉], Xiukun Wang[3,6], Safia Malki[3], Yanpui Chan[1], Brian Bennett[3], Joshua Feng[4], Jiaqi Tang[4], Xi Chen[1], Daniel McKim[1], Chao Zhang[4], Litao Tao[5], Jie Xu[2], Y. Eugene Chen[2], Guang Hu[3✉] & Qi-Long Ying[1✉]

[1]Eli and Edythe Broad Center for Regenerative Medicine and Stem Cell Research at USC, Department of Stem Cell Biology and Regenerative Medicine, Keck School of Medicine, University of Southern California, Los Angeles, CA 90033, USA

[2]Center for Advanced Models Translational Sciences and Therapeutics, University of Michigan Medical School, 2800 Plymouth Road, Ann Arbor, MI 48109, USA

[3]Epigenetics and RNA Biology Laboratory, National Institute of Environmental Health Sciences, RTP, NC 27709, USA

[4]Loker Hydrocarbon Research Institute & Department of Chemistry, University of Southern California, Los Angeles, CA 90089, USA

[5]Department of Biomedical Sciences, School of Medicine, Creighton University, Omaha, NE 68178, USA

[6]These authors contributed equally

✉Email: wangduotc@gmail.com (D.W.); guang.hu@nih.gov (G.H.); qying@med.usc.edu (Q.-L.Y.).

Materials and Methods

Mice

Adult female mice were used in these experiments. Embryos for cell line derivation were obtained from B6D2F1 mice. C57BL/6J mice were used as host embryos for chimera generation, and ARC mice served as recipients for embryo transfer. All procedures involving ESC injection into blastocysts, blastocyst transfer, and post-transfer animal care were performed at the Irvine Transgenic Mouse Facility, University of California, Irvine.

All animal studies were conducted in accordance with institutional guidelines for the care and use of laboratory animals and were approved under IACUC protocol #AUP-22-126. Experiments related to the collection of mouse embryos for ESC derivation were performed at the Department of Animal Resources, University of Southern California. The project received approval from the Animal Welfare and Ethical Review Bodies at both the University of California, Irvine and the University of Southern California.

Cell Cultures

Cell lines used in this study are listed in the Key Resources Table. All cells were cultured in the incubators at 37°C with 5% CO_2, without the use of antibiotics.

Conversion of ESCs to Formative cells

ESCs were initially plated in AloXR medium, consisting of N2B27 supplemented with 3 ng/mL Activin A, 2 μM XAV939, and 1.0 μM BMS493, as previously described.[1] Complete conversion typically requires approximately 4–5 passages. After conversion, the cultures were maintained at higher densities and passaged using Accutase.

Conversion of ESCs to EpiSCs

ESCs were initially plated in AFX medium, consisting of N2B27 or DMEM supplemented with 10% FBS and containing 20 ng/mL Activin A, 20 ng/mL bFGF, and 2 μM XAV939. Complete conversion typically required approximately 4–5 passages.

Derivation of ESCs from mouse blastocyst

E3.5 B6D2F1 mouse blastocysts were flushed from the oviduct. The zona pellucida was removed using acid Tyrode's solution, after which blastocysts were transferred onto feeder-coated culture plate. Embryos were cultured in N2B27 medium supplemented with either BRD0705/PD03 or CHIR/PD03. After approximately 4-5 days, the outgrowths were dissociated into single cells using 0.025% trypsin and passaged for further expansion.

Western blot

The cells were lysed using ice-cold RIPA buffer, comprising 1% Triton X-100, 50 mM Tris, 150 mM NaCl, 0.1% SDS, and 1% sodium deoxycholate, with protease and phosphatase inhibitors (Roche) added. After denaturation at 95°C, the proteins, mixed with Laemmli sample buffer (Bio-Rad), were separated on a 10-15% polyacrylamide gel and transferred onto a PVDF

membrane (Millipore) via electrophoresis. The membrane was rinsed with TBST (0.1% Tween-20 in TBS), blocked with 5% Blocker (Bio-Rad) in TBST for 1 hour at room temperature, then incubated with primary antibodies at 4°C overnight, followed by a 1-hour incubation with HRP-conjugated secondary antibodies at room temperature the following day.

Quantitative real-time PCR (qRT-PCR)

Total RNA was isolated using the RNeasy Mini Kit (Qiagen) following the manufacturer's instructions. Complementary DNA (cDNA) synthesis was carried out with the iScript cDNA Synthesis Kit (Bio-Rad). Quantitative real-time PCR was conducted using the iTaq Universal SYBR® Green Supermix (Bio-Rad) on a Viia 7 real-time PCR system. Gene expression levels were normalized to Gapdh.

Chemical Screening

A focused small-molecule library containing 164 compounds (Supplementary information, Table S1) was screened using ESCs and EpiSCs to identify novel regulators of pluripotent stem cell self-renewal and to gain insights into their underlying mechanisms. ESC self-renewal and to gain insights into their underlying mechanisms. The library consisted of compounds targeting key regulators of cell proliferation and stemness-associated signaling pathways.

In the primary screen, ESCs cultured in DMEM supplemented with 10% FBS were treated with individual compounds and compared with untreated controls. After 4 days of treatment, 47 compounds were found to enhance ESC colony formation. From these candidates, five top-ranking and previously unreported compounds that produced the largest increases in colony number were selected for further validation. Their ability to promote ESC self-renewal in the absence of 2i/LIF was subsequently assessed by alkaline phosphatase (AP) staining.

ESCs treated with each of the five candidate compounds were then passaged over an extended period to evaluate long-term self-renewal. Among them, BRD0705 was the only compound that consistently maintained ESC self-renewal during prolonged culture. OCT4-GiP ESCs[2] cultured under LIF/serum conditions were initially used to confirm the ability of BRD0705 to support ESC self-renewal. Quantitative RT–PCR analyses were subsequently performed to assess the expression of pluripotency marker genes following long-term expansion with BRD0705 alone, and the capacity of these cells to revert to naïve ESC culture conditions (2iL) was evaluated.

For EpiSC experiments, the same small-molecule library (Supplementary information, Table S1) was screened in parallel using mouse EpiSCs cultured in DMEM supplemented with 10% FBS. Differences between treated and untreated cells were assessed based on colony morphology and colony number after 4 days of treatment. This screen identified 21 compounds that promoted EpiSC self-renewal (Supplementary information, Fig. S2a).

TopFlash Luciferase Assay

The luciferase reporter plasmid pB-TopFlash-NanoLucP was obtained from Addgene. This construct, together with the hyPBase transposase plasmid, was co-introduced into both WT and β-catenin$^{-/-}$ ESCs using Lipofectamine™ LTX Reagent with PLUS™ Reagent (Thermo

Fisher Scientific), according to the manufacturer's instructions. Transfected cells were seeded into 24-well plates treated with either CHIR99021 or BRD0705 in N2B27 medium for 24–36 hours. Following treatment, the culture medium was removed and the cells were rinsed with PBS. Luciferase activity was measured using the Nano-Glo® Dual-Luciferase® Reporter Assay System (Promega) according to the manufacturer's protocol. Luminescence was detected using a GloMax® Explorer Multimode Microplate Reader.

ESCs culture

ESCs were cultured on 0.1% gelatin-coated dishes at 37°C in a 5% CO_2 atmosphere. DMEM/10% FBS medium was prepared by supplementing DMEM (Gibco) with 10% FBS (Gibco), 0.1 mM β-mercaptoethanol (Sigma), 1% MEM Non-Essential Amino Acids Solution (Gibco), and 2 mM L-glutamine (Gibco).

DMEM/F12-N2 medium was prepared by adding 1 ml of N2 (Gibco) 100× stock solution to 100 ml of DMEM/F12. Neurobasal/B27 medium was prepared by supplementing Neurobasal medium with 2 mL of B27 supplement (Gibco) and 2 mM L-glutamine per 100 mL. N2B27 medium was generated by mixing DMEM/F12-N2 and Neurobasal/B27 media at a 1:1 ratio, followed by the addition of 0.1 mM β-mercaptoethanol.

To maintain naïve-state ESCs, 3 μM CHIR99021 (Selleckchem), 1 μM PD0325901 (PD03; Selleckchem), and 10 ng/mL LIF (PeproTech) (2iL) were added to either DMEM/10% FBS or N2B27 medium. E14TG2a ESCs could be maintained under 2iL conditions in either DMEM/10% FBS or N2B27 medium, with or without feeder cells. In contrast, B6D2F1 ESCs required feeder-coated plates and were cultured in 2iL/N2B27 or 2iL/DMEM/10% FBS medium.

For BRD0705/IWR conditions, 8 μM BRD0705 (Cayman Chemical) and 2.5 μM IWR-1 (Selleckchem) were added to either DMEM/10% FBS or N2B27 medium. Under BRD0705-only or BRD0705/IWR-1 conditions, ESCs required feeder cells when cultured in N2B27 medium, whereas feeder cells were not required in DMEM/10% FBS, where 0.1% gelatin coating was sufficient.

β-catenin–deficient ESCs ($Ctnnb1^{-/-}$)[3,4] were cultured on γ-irradiated CF-1 mouse embryonic fibroblast (MEF) feeders in N2B27 medium containing 20 ng/ml LIF and 1 μM PD03 (Selleckchem).

EpiSCs culture

CD1 EpiSCs were established from the epiblasts of E5.75 CD1 mouse embryos, as previously described.[3] To generate E14TG2a-EpiSC, E14TG2a-ESC were cultured in a basal medium containing 20 ng/ml Activin A (PeproTech), 20 ng/ml bFGF (PeproTech), and 2 μM XAV-939 (Sigma).

Both CD1 and E14TG2a-EpiSC were subsequently maintained in DMEM/10%FBS medium or N2B27 medium with the addition of 1.5 μM CHIR99021 and 2.5 μM IWR-1. When using N2B27 medium, culturing EpiSCs on feeder cells is more effective. However, when using

DMEM/FBS medium, feeder cells are not required, and pre-coating the culture plates with 0.1% gelatin is sufficient.

Chimeras Generation

Approximately 15 dissociated single cells were injected into each blastocyst-stage embryo. Injected embryos were subsequently transferred into pseudo-pregnant recipient mice. Imaging of E10.5 mid-gestation embryos was performed using a Keyence BZ-X800 fluorescence microscope. For isolation of primordial germ cells (PGCs) from chimeric embryos, embryos were harvested at embryonic day 15.5 (E15.5).

Embryoid Body Formation and Differentiation

A total of 2×10^4 cells were seeded into AggreWell™400 (STEMCELL Technologies) using either N2B27 medium or IMDM/FBS medium[5] (IMDM supplemented with 15% fetal bovine serum, 2 mM L-glutamine, 0.05 mg/mL ascorbic acid, and 0.001% monothioglycerol). After 2–3 days of incubation, the resulting embryoid bodies (EBs) were transferred onto laminin- or gelatin-coated plates with fresh medium for outgrowth. For neural induction, ESCs or EpiSCs were plated on laminin-coated plates in N2B27 medium.[6,7] For mesoderm induction, ESCs or EpiSCs were plated on gelatin-coated plates in IMDM/FBS medium[5]. For endoderm induction, ESCs or EpiSCs were plated on gelatin-coated plates in either IMDM/FBS or GMEM supplemented with 10% FBS.

Derivation of Sox1 positive mouse Neural Stem Cells

Neural differentiation was performed using Sox1–GFP–puromycin resistance knock-in mouse ESCs (46C line) through an adherent monolayer culture system in N2B27 medium, as previously described[8]. N2B27 consisted of a 1:1 mixture of DMEM/F12 and Neurobasal media, supplemented with 0.5× N2, 0.5× B27, 1% L-glutamine, and 0.1 mM β-mercaptoethanol. Under these serum-free conditions, Sox1-GFP positive, puromycin-resistant Rosette NSCs appeared between days 5 and 7. A transient selection with 1.0 μg/ml puromycin, followed by fluorescence-activated cell sorting (FACS) using the BD FACSAria III system, effectively eliminated Sox1-negative cells, resulting in a highly enriched population of Sox1-GFP positive mouse NSCs at Passage 0. Purified Sox1–GFP–positive NSCs were subsequently cultured as neurospheres in non-adherent Petri dishes using N2B27 medium supplemented with bFGF/EGF.

Flow cytometry analysis

ESC (RFP-Zeocin) and CD1-EpiSC (GFP-IP) were dissociated using 0.025% trypsin (GIBCO). Following dissociation. After a single wash with PBS, the cells were resuspended in PBS containing 2% fetal bovine serum with DAPI for analysis. Finally, the stained cells were analyzed using a flow cytometer.

Immunofluorescence analysis

Cells were fixed on plates with 4% paraformaldehyde (PFA) for 15 minutes at room temperature (RT), followed by three washes with PBS. Blocking was performed using 5% BSA

in PBS containing 0.3% Triton X-100 for 1 hour. Primary and secondary antibodies were incubated either for 1 hour at RT or overnight at 4°C, with antibody dilutions prepared in PBS containing 1% BSA and 0.3% Triton X-100. Antibodies used in this study are listed in the Key Resources Table. Fluorescence images were acquired using Keyence BZ-X800 fluorescence microscope.

Alkaline phosphatase (AP) staining

The AP staining reagent was prepared with 200 mM Tris-HCl buffer (pH 8.2) according to the manufacturer's instructions. Cells were washed with PBS and then incubated in the AP staining reagent (Vector Laboratories, SK-5300) for 30 minutes at room temperature in a darkroom. Following incubation, the cells were rinsed with PBS and fixed with 4% formaldehyde at 4°C overnight. After two PBS washes, the cells were observed using Keyence BZ-X800 fluorescence microscope.

Single-cell RNA-seq

Co-cultured cells were trypsinized and dissociated into a single-cell suspension in DMEM supplemented with 10% FBS. Single-cell RNA sequencing was performed according to the manufacturer's instructions using the 10x Genomics Chromium Next GEM Single Cell 3′ Kit v3.1

Cut&Tag

For CUT&Tag analysis, co-cultured ESC- and EpiSC-derived cells were separated by fluorescence-activated cell sorting (FACS) based on GFP and RFP expression. A total of 2 × 10^5 cells per sample were used for CUT&Tag targeting H3K4me3, H3K27me3, and H3K27ac, using the CUTANA™ CUT&Tag Kit (Epicypher) according to the manufacturer's instructions. Libraries were sequenced on Illumina platforms.

Single-cell analysis

For co-cultured cells analyzed in this study, single-cell RNA-seq FASTQ files were aligned to the mouse reference genome (mm39) and processed into barcoded count matrices using 10X Genomics cellranger 7.2 software. GFP and RFP sequences were added to the mm39 genome to identify cells with GFP and RFP expression, respectively. Single-cell count matrices were analyzed using Seurat version 4.3. Prior to SCT transformation, cells with less than 4000 features, less than 10000 counts, and greater than 10% mitochondrial RNA were removed. A total of 4225 cells remained after filtering. Gene expression UMAP plots (Supplementary information, Fig. S5b and c) were created using the Seurat FeaturePlot function. Naïve cell markers were Nanog, Esrrb, Zfp42, Nr0b1, Tfcp2l1, Tbx3, Tcl1 and Prdm14. Primed cell markers were Fgf5 and Pitx2. Vimentin was used as a MEF cell marker. A UMAP showing the 6 unique cell clusters was created with the Seurat DimPlot function (Supplementary information, Fig. S5d). For public single cell RNA-seq data, raw FASTQ files were downloaded from GEO (GSE45719, GSE100597 and GSE74155). Gene read counts (TPM normalized) were obtained using Salmon v0.14.1 and GENCODE vM24.

For the UMAP plot in Fig.1m, counts for all cells in the plot were combined into one matrix, and quantile normalization was performed using the “normalize.quantiles” function from the “preprocessCore” R package. Batch correction was performed using the “removeBatchEffect” function from the “limma” R package. The UMAP plot with the merged data sets was created using the “umap” R package. First, the UMAP base was generated using the “umap” function and samples from GSE45719 and GSE100597. Next, the additional samples, including ESCs and EpiSCs from GSE74155, and our co-cultured cells, were added using the “predict” function. For the correlation plot in Fig. S5e and f, gene read counts (TPM normalized) were obtained using Salmon v0.14.1 and GENCODE vM24 for additional bulk RNA-seq samples (GSE131553) and combined with the single cell counts (this study and GSE45719, GSE100597, GSE74155). These counts were then processed and normalized in the same way as for the UMAP plot. After this, genes were filtered to only include the top 2,000 genes with the highest average expression. The Spearman correlation was calculated using the average expression across cells for each cell type.

RNA-seq data analysis

For public RNA-seq data, Raw FASTQ files for public RNA-seq data were downloaded from GEO (GSE45719, GSE100597, GSE74155, and GSE131553). Gene read counts (TPM normalized) were obtained using Salmon v0.14.1 and GENCODE vM24. All data sets were merged, and quantile normalization was performed using the “normalize.quantiles” function from the “preprocessCore” R package. Batch correction was performed using the “removeBatchEffect” function from the “limma” R package. The UMAP plot with the merged data sets was created using the “umap” R package. First, the UMAP base was generated using the “umap” function and samples from GSE45719[9] and GSE100597[10]. Next, the additional samples were added using the “predict” function. For the correlation plot, genes were first filtered to only include the top 2,000 genes with the highest average expression. Next, the Spearman correlation was calculated using the average expression across cells for each cell type.

Bulk RNA-seq analysis

For bulk RNA-seq data generated in this study and from GSE131556, the raw reads were trimmed with cutadapt (v3.7) first, and the filtered reads were then aligned to mm9 reference with STAR (v2.7.0b). Differentially expressed genes were identified using DESeq2 v1.40.2 and required an FDR of less than 0.05 and a fold change of two or greater. The expression heatmap was generated using the “heatmap.2” function from the “plots” R package. The average expression was Z-score normalized for each gene. The heatmap only used genes that were differentially expressed in both EpiSC compared to 2iESC and also scPrime compared to scNaïve.

ChIP-seq and Cut&Tag data analysis

For public ChIP-seq data, raw FASTQ files were downloaded from GEO (GSE156261). ChIP-seq and CUT&Tag-seq samples were processed using the same pipeline described here. Reads were filtered to only include those with a mean PHRED quality score of 20 or greater. Adapter was trimmed from reads using Cutadapt v4.5. Reads were aligned using Bowtie 2

v2.5.2 and the mm10 reference genome with parameters: “--local --very-sensitive --no-mixed --no-discordant”. Duplicate reads were removed using the “MarkDuplicates” tool from the Picard Tools suite v3.1.1. Read coverage was derived from the fully aligned fragments using the “genomecov” tool from the bedtools suite v2.31.1. Read coverage was normalized to depth per 10 million aligned reads. Peaks were called using MACS2 v2.2.9.1. Naïve-specific peaks were defined as peaks with at least 4 times as many depth-normalized reads in the Naïve sample as the Primed sample (and vice versa for Primed-specific peaks). Heatmaps were generated using deepTools v3.5.4. Bivalent genes were identified by finding TSSs (from GENCODE vM24.basic) with both a H3K4me3 and a H3K27me3 peak within 2 kb of the TSS.

Quantification and Statistical Analysis

All data are presented as mean ± SEM. A two-tailed Student's t-test was employed to assess statistical significance, with error bars representing the SEM of three independent experiments. A P-value of less than 0.05 was considered statistically significant. Statistical significance is indicated as follows: *$P < 0.05$, **$P < 0.01$, and ***$P < 0.001$.

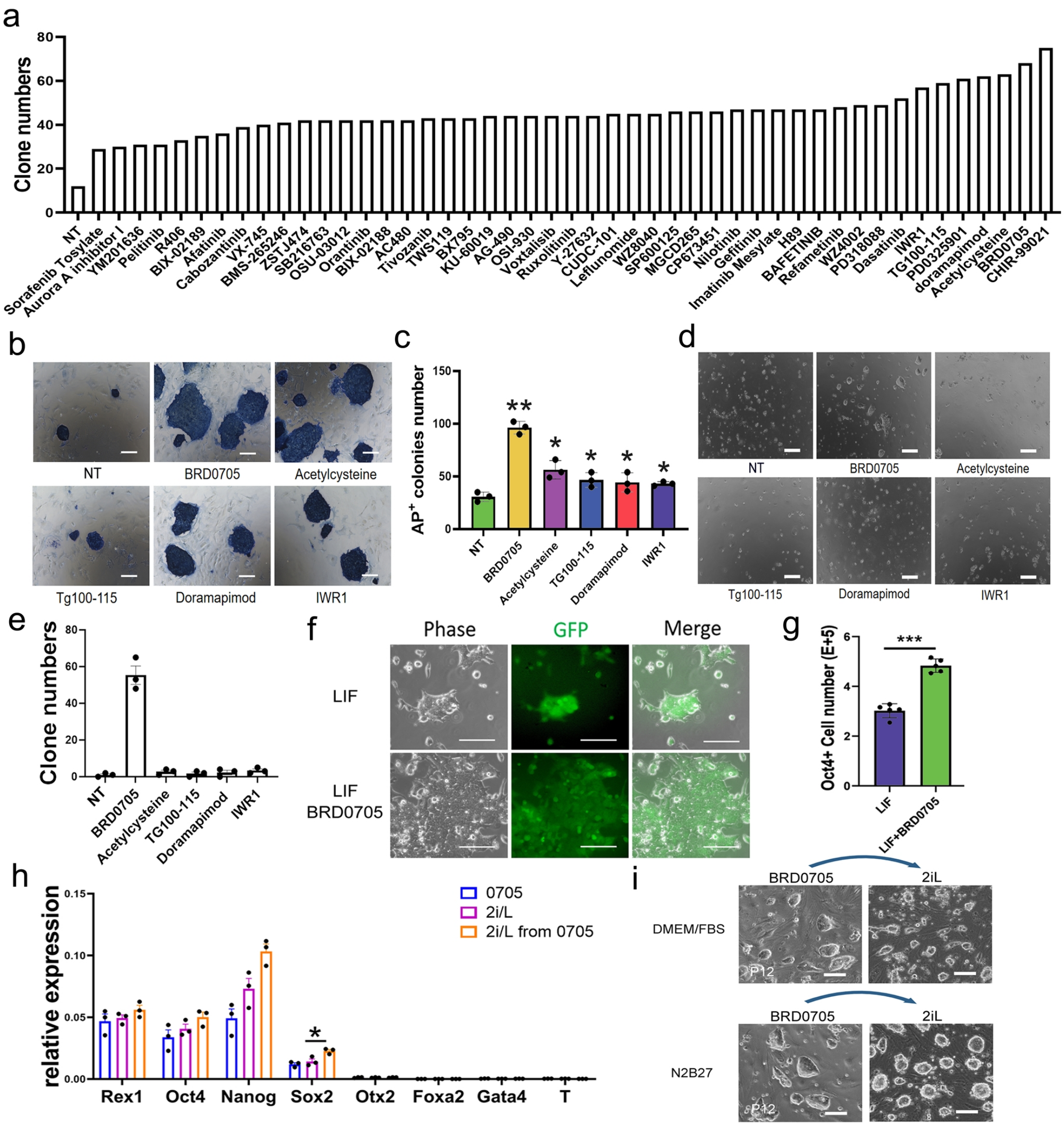

Supplementary information, Fig. S1. BRD0705 promotes ESC self-Renewal.

a Bar graph summarizing the results of a small-molecule screen performed in mouse ESCs. Only compounds that enhanced ESC colony formation relative to the no-treatment (NT) control are shown.

b Alkaline phosphatase (AP) staining of ESC colonies formed under treatment with the five selected compounds from the smal-molecule library screen. Cells were cultured in DMEM/FBS medium for 4 days. Scale bar, 100 µm. NT, no-treatment control.

c Quantification of AP+ colonies in b. Data represent mean ± SEM. from triplicate experiments. *, $p<0.05$, indicating a statistically significant difference in the number of AP+ clones compared with the no-treatment control under the indicated inhibitor treatments.

d The cellular morphology of ESC treated with the five selected compounds from the small-molecule library screen. Cells were at passage 3 under the above conditions, cultured in DMEM/FBS. Scale bars, 200 µm.

e Quantification of d, showing numbers of ESC colonies formed under the indicated treatment conditions.

f Representative phase-contrast and fluorescence images of Oct4-GiP ESCs cultured in LIF alone or with BRD0705.The basal cell culture medium was DMEM/10%FBS. Scale bar, 100 μm.

g Quantification of Oct4-GFP$^+$ colony ratio from the experiments shown in f. Data are presented as mean ± SEM from three biological replicates. *, $p<0.05$. **, $p<0.01$. ***, $p<0.001$.

h qRT-PCR analysis of pluripotency and germ layer-specific gene expression in ESCs maintained in 2iL, BRD0705, or in cells transitioned from BRD0705 to 2iL, normalized to GAPDH. Data are presented as mean ± SEM from three biological replicates *, $p<0.05$.

i Left panel: Phase-contrast images of Passage 12 B6D2F1 ESCs (derived from offspring of BDA male mice crossed with C57BL/6 female mice) cultured in BRD0705 with either N2B27 or DMEM/FBS medium. Right panel: Morphology of ESCs after 12 passages in N2B27 or DMEM/FBS medium with BRD0705, followed by a switch to 2iL conditions for three additional passages. Scale bars, 100 μm.

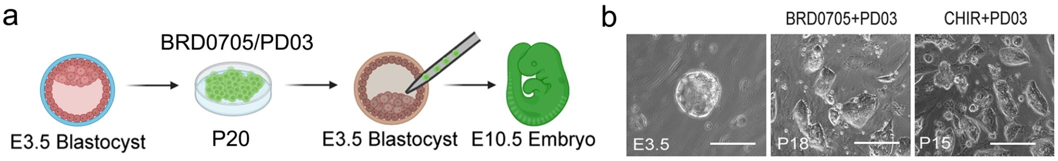

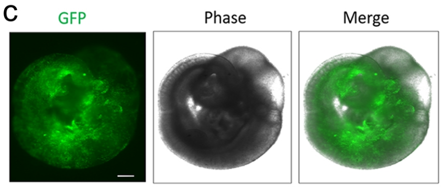

d

	Embryos recovered	E10.5 chimeric embryos	%E10.5 chimeric embryo
ESC(BRD0705+PD03)	7	4	57.1%

Supplementary information, Fig. S2. ESCs derived under the BRD0705/PD03 condition retain chimera-forming competence after long-term culture.

a Schematic illustration of the chimera formation assay. ESCs derived from E3.5 blastocysts and maintained for 20 passages (P20) under the BRD0705/PD03 condition were microinjected into E3.5 recipient blastocysts, which were subsequently transferred into surrogate females for development to E10.5 embryos.

b Phase-contrast images of mouse blastocysts (E3.5), passage 18 ESCs derived under BRD0705+PD03 conditions, and passage 15 ESCs cultured under CHIR+PD03 conditions. Scale bar: 200 μm.

c Bright-field and fluorescent images of E10.5 mouse embryos generated after blastocyst injection of BRD0705+PD03 cultured ESCs with ubiquitous GFP. Scale bars, 350 μm.

d Summary of chimera experiments at E10.5.

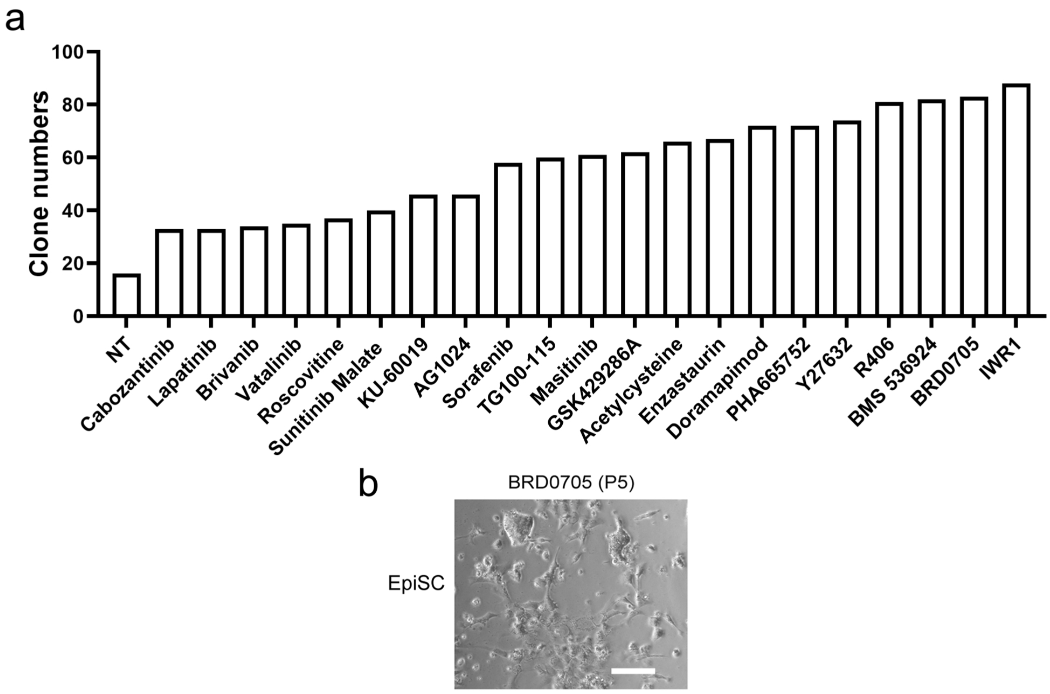

Supplementary information, Fig. S3. BRD0705 supports EpiSC self-renewal.

a . Bar graph showing the results of a small-molecule screen conducted in EpiSCs. Only compounds that increased colony numbers relative to the control condition are shown.

b . Representative images showing the morphology of EpiSC after five passages in DMEM/FBS/BRD0705 culture condition. Scale bars, 100 μm.

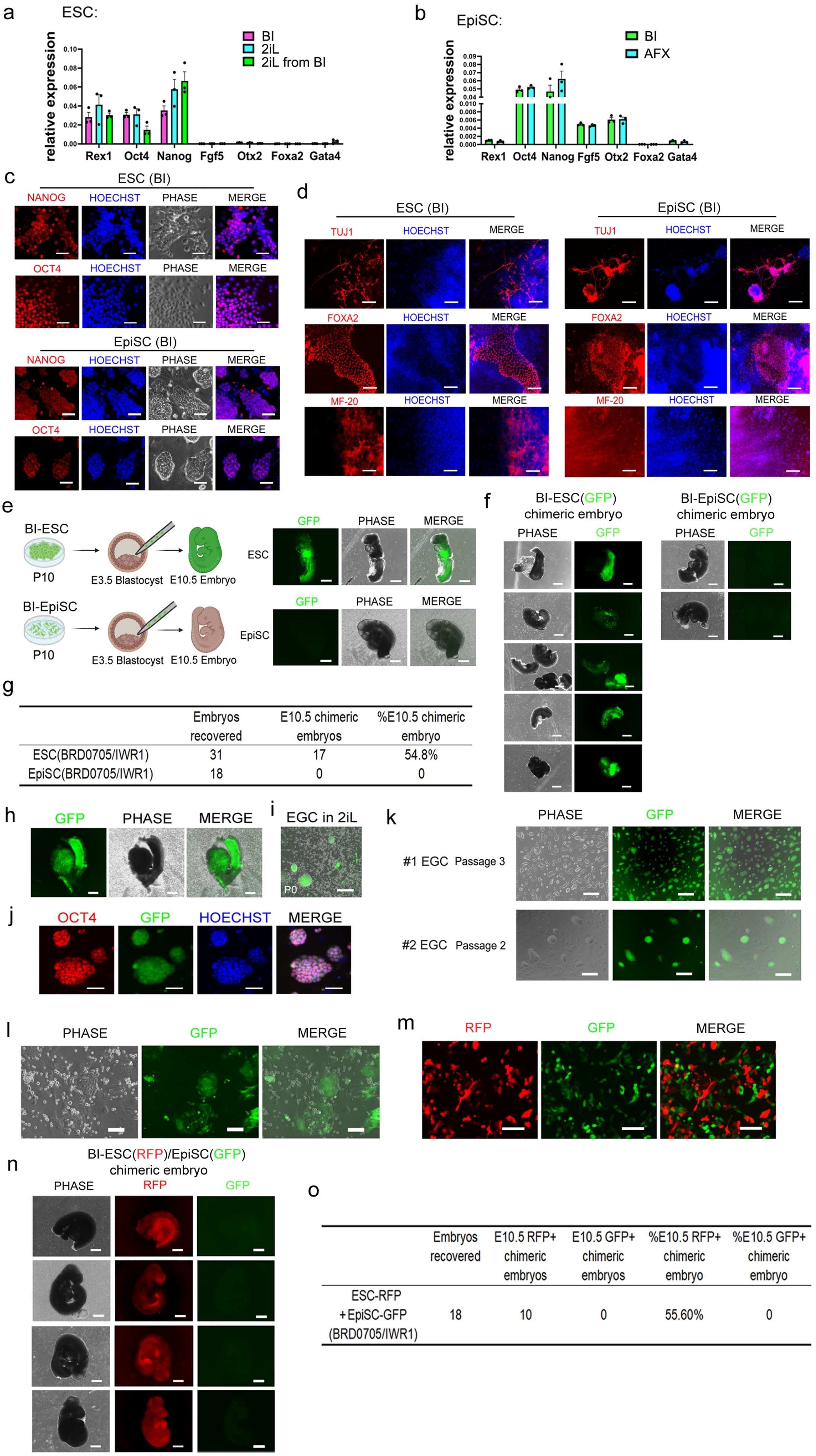

g

	Embryos recovered	E10.5 chimeric embryos	%E10.5 chimeric embryo
ESC(BRD0705/IWR1)	31	17	54.8%
EpiSC(BRD0705/IWR1)	18	0	0

o

	Embryos recovered	E10.5 RFP+ chimeric embryos	E10.5 GFP+ chimeric embryos	%E10.5 RFP+ chimeric embryo	%E10.5 GFP+ chimeric embryo
ESC-RFP +EpiSC-GFP (BRD0705/IWR1)	18	10	0	55.60%	0

Supplementary information, Fig. S4. Characterization of ESC and EpiSC identity maintained under the BI condition.

a qRT-PCR analysis of marker gene expression in ESCs cultured in BI for 15 passages, 2iL, or in 2iL following transition from BI-cultured ESCs. Expression levels were normalized to GAPDH. Data represent mean ± SEM, n=3.

b qRT-PCR analysis of marker gene expression in EpiSCs treated with BI, ActivinA, bFGF, or IWR1. Expression levels were normalization to GAPDH. Error bars represent SEM from technical triplicates(n=3).

c Immunofluorescence images showing expression of the pluripotency markers NANOG and OCT4 in ESCs and EpiSCs cultured under the BI condition for 12 passages. Scale bar: 50 μm.

d IF images demonstrate three germ layers differentiation potential of ESC and EpiSC cultured in BRD0705/IWR-1, showing differentiation into endoderm (FOXA2), mesoderm (MF-20), and ectoderm (TUJ1). Scale bar: 250 μm

e Schematic representation of the experimental workflow showing the injection of P10 BI-expanded ESCs or EpiSCs into E3.5 blastocysts, followed by embryo development to E10.5 (Left). Representative phase-contrast and fluorescence images of E10.5 mouse embryos derived from blastocyst injection of GFP-expressing ESCs or EpiSCs cultured with BI (right). Scale bars, 500 μm.

f Representative phase-contrast and fluorescence images of E10.5 mouse embryos derived from WT blastocyst injection of GFP-labeled BI-ESC or GFP-labeled BI-EpiSC. Scale bars, 500 μm.

g Summary of chimera experiments at E10.5.

h Phase-contrast and fluorescence images of gonads originating from E15.5 mouse embryos obtained from blastocyst injection of ESCs-GFP cultured with BI. Scale bars, 350 μm.

i Phase-contrast and fluorescence images show #1 embryonic gonad cells (EGCs) derived from the gonads of E15.5 chimeric embryos formed by ESCs-GFP cultured in BI. EGCs cultured under 2iL conditions. Scale bars, 250 μm.

j IF analysis verifies the expression of GFP and OCT4 in EGCs cultured in 2iL. Scale bar 50μm.

k Phase-contrast and fluorescence images of EGCs derived from two different gonads (gonads obtained from two E15.5 chimeric mouse embryos produced via wild-type blastocyst injection of GFP-labeled ESC cultured with BI), with cells cultured under 2i/LIF conditions. Scale bars, 250 μm. #1 and #2 represent EGCs derived from the gonads of two distinct chimeric mice.

l Representative phase-contrast and fluorescence images of mixed cells derived from E10.5 mouse embryos obtained from WT blastocyst injections of GFP-expressing ESC cultured with BI. The image shows beating GFP-positive muscle cells, with a video of the beating cells provided in Supplementary Movie S1. Scale bars, 100 μm.

m The phase-contrast and fluorescence images of Passage 1 ESC (RFP^{+})/EpiSC (GFP^{+}) cultured in N2B27 medium supplemented with BRD0705/IWR-1, respectively. Scale bars, 100 μm.

n Representative images of phase-contrast and fluorescence microscopy of E10.5 mouse embryos following blastocyst injection with a 1:1 mixture of RFP-labeled ESC and GFP-labeled EpiSC, cultured in the presence of BI. Scale bars, 500 µm.

o Summary of chimera experiments at E10.5.

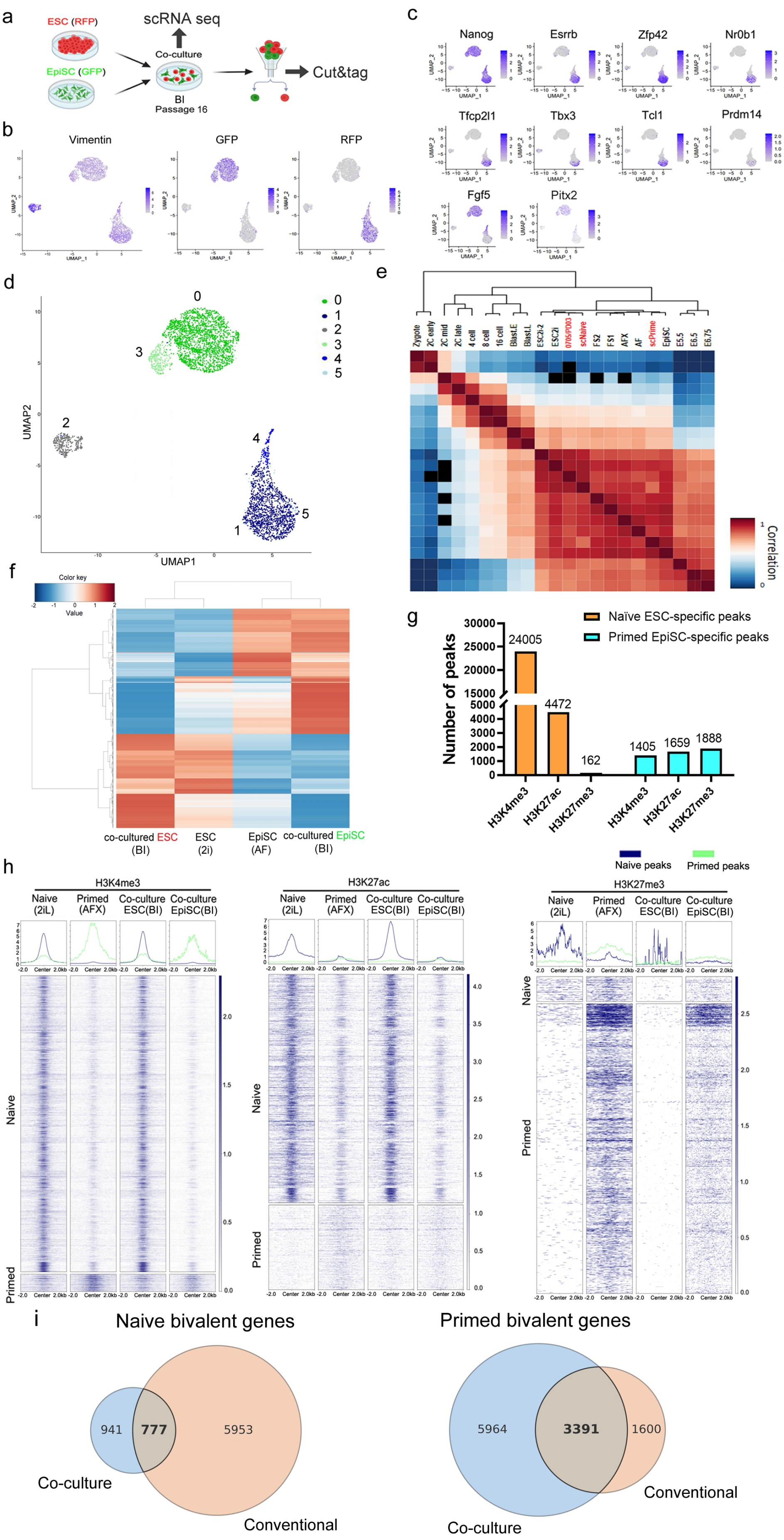
a
ESC (RFP)
EpiSC (GFP)
scRNA seq
Co-culture
BI
Passage 16
Cut&tag
b
Vimentin
GFP
RFP
c
Nanog
Esrrb
Zfp42
Nr0b1
Tfcp2l1
Tbx3
Tcl1
Prdm14
Fgf5
Pitx2
d
UMAP1
UMAP2
0
1
2
3
4
5
e
Zygote
2C early
2C mid
2C late
4 cell
8 cell
16 cell
Blast.E
Blast.L
ESC2i-2
ESC2i
0705PD03
scNaive
FS2
FS1
AFX
AF
scPrime
EpiSC
E5.5
E6.5
E6.75
Correlation
f
Color key
Value
co-cultured ESC (BI)
ESC (2i)
EpiSC (AF)
co-cultured EpiSC (BI)
g
Naïve ESC-specific peaks
Primed EpiSC-specific peaks
Number of peaks
24005
4472
162
1405
1659
1888
H3K4me3
H3K27ac
H3K27me3
h
Naive peaks
Primed peaks
H3K4me3
H3K27ac
H3K27me3
Naive (2iL)
Primed (AFX)
Co-culture ESC(BI)
Co-culture EpiSC(BI)
Naive
Primed
i
Naive bivalent genes
941
777
5953
Co-culture
Conventional
Primed bivalent genes
5964
3391
1600
Co-culture
Conventional

Supplementary information, Fig. S5. scRNA-seq and chromatin landscape analysis of ESC/EpiSC co-cultured under BI conditions.

a Schematic illustration of the experimental design. ESCs (RFP^+) and EpiSCs (GFP^+) were co-cultured under BI conditions for 16 passages, followed by scRNA-seq and Cut&tag analyses.

b UMAP plots displaying the expression of Vimentin, GFP, and RFP confirm the presence of ESCs and EpiSCs in co-culture under BI conditions.

c UMAP visualization of key pluripotency-related genes, including the PSC marker Nanog; ESC markers (*Esrrb, Zfp42, Nr0b1, Tfcp2l1, Tbx3, Tcl1, and Prdm14*); and the EpiSCs marker *Fgf5* and *Pitx2*, highlights the transcriptional differences between ESCs and EpiSCs.

d UMAP clustering of single-cell transcriptomes reveals distinct cell populations within the co-culture cells, labeled as clusters 0–5.

e Heatmap showing the transcriptomic correlation between co-cultured cells under BI conditions and reference ESCs, EpiSCs, and formative cells (fs) cultured under different conditions, indicating their resemblance to ESC and EpiSC pluripotent states. scNaïve: single-cell sequencing results of ESCs in BI co-cultured cells. scPrime: single-cell sequencing results of primed PSCs in BI co-cultured cells. sc RNA-seq reference data for embryonic developmental stages were obtained from datasets GSE45719 and GSE100597. The reference data for ESCs, FS cells, and EpiSCs indicated in black font were obtained from the dataset GSE131556.

f Heatmap showing the transcriptomic correlation of ESC and EpiSC co-cultured under BI conditions compared to reference ESC (2i) and EpiSC (AF).[11] The gene expression profiles of co-cultured ESCs and EpiSCs closely resemble those of ESCs cultured in 2i and EpiSCs cultured in AFX, respectively, indicating that BI maintains the transcriptional identity of both cell types.

g Bar graph quantifying the number of naïve ESCs-specific and primed EpiSCs-specific chromatin accessibility peaks for H3K4me3, H3K27ac, and H3K27me3 modifications.

h Heatmaps displaying Cut&Tag profiles for H3K4me3, H3K27ac, and H3K27me3 modifications in naïve ESCs (2iL), primed EpiSCs (AFX), and BI co-cultured ESCs (RFP^+) and EpiSCs (GFP^+). Naïve ESCs exhibit distinct chromatin accessibility patterns compared to primed EpiSCs, and co-cultured ESCs and EpiSCs maintain their respective epigenetic signatures, confirming the long-term maintenance of their identities under BI conditions.

i Venn diagrams illustrating the overlap between bivalent genes identified in ESCs and EpiSCs co-cultured under BI conditions and those reported in ESCs and EpiSCs under conventional culture conditions. The left diagram represents naïve bivalent genes, showing 777 genes shared between co-cultured and conventional ESCs. The right diagram depicts primed bivalent genes, highlighting 3,391 genes common to co-cultured primed ESCs and conventional EpiSCs. Reference data for bivalent gene comparisons were obtained from the publicly available dataset GSE156261.

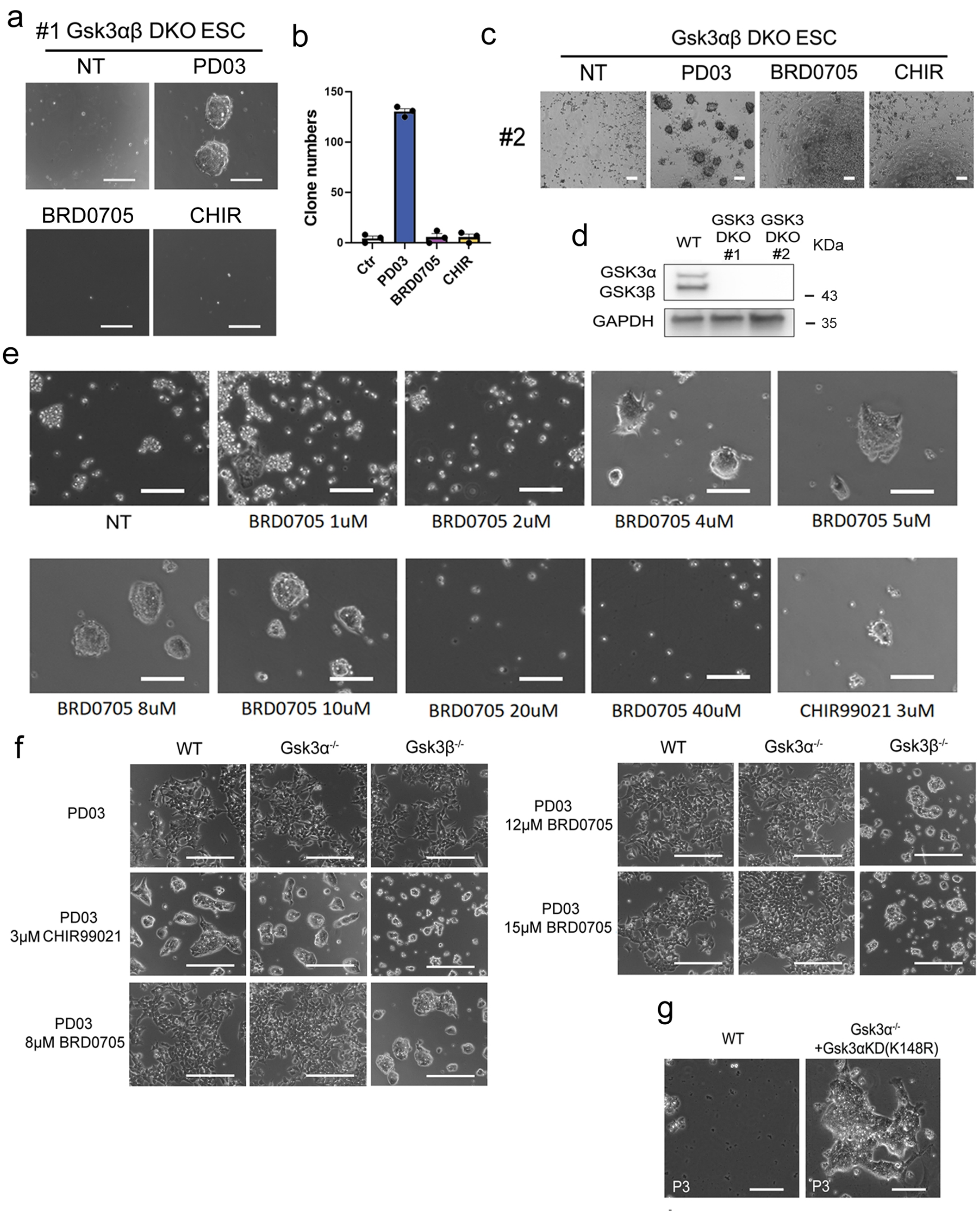

Supplementary information, Fig. S6. BRD0705 regulates ESC self-renewal through Gsk3α inhibition independent of β-Catenin

a Morphological comparison of the #1 Gsk3αβ DKO ESC line under the indicated treatment conditions. Cells were cultured under non-treatment control, PD03, BRD0705, or CHIR conditions in DMEM/FBS medium for three Passage. Scale bars ,100 μm.

b Quantification of colony numbers corresponding to the images in **a**. PD03 treatment significantly increased colony formation in Gsk3α/β DKO ESCs compared with the no-treatment control and other conditions (BRD0705 and CHIR), which showed minimal colony formation. Error bars indicate mean ± SEM.

c Morphological comparison of Gsk3α/β DKO ESC clones under different culture conditions. Representative images of the #2 Gsk3α/β DKO ESC line cultured in DMEM/FBS medium under no-treatment (NT), PD03, BRD0705, or CHIR conditions for three passages. Scale bars, 100 μm.

d Western blot analysis of GSK3α and GSK3β in wild-type (WT) ESCs and in two independent GSK3α/β double-knockout (DKO) ESC lines (#1 and #2). GSK3α and GSK3β were detected in WT cells but were absent in both DKO lines, confirming successful knockout. GAPDH served as a loading control.

e Dose-dependent effects of BRD0705 on self-renewal of Gsk3β$^{-/-}$ ESCs. Representative images show Gsk3β$^{-/-}$ ESCs cultured in N2B27/PD03 medium with increasing concentrations of BRD0705 (1–40 μM) for two passages, compared with CHIR99021 (3 μM). Scale bars, 100 μm.

f Morphological analysis of WT, Gsk3α$^{-/-}$, and Gsk3β$^{-/-}$ ESC cultured in N2B27 medium with PD03 alone or in combined with CHIR99021 or BRD0705 (8 μM, 12 μM, and 15 μM) for three passages. Representative images inlustrate differences in colony morphology and cell density. Scale bars: 200 μm.

g Representative images showing the morphology of wild-type (WT) ESCs and Gsk3α$^{-/-}$ ESCs expressing a kinase-dead Gsk3α (K148R) mutant at passage 3 (P3). Cells were cultured in DMEM/FBS medium for three passages. By passage 3, all WT ESCs had died, whereas Gsk3α$^{-/-}$ ESCs expressing kinase-dead Gsk3α retained colony. Scale bars represent 100 μm.

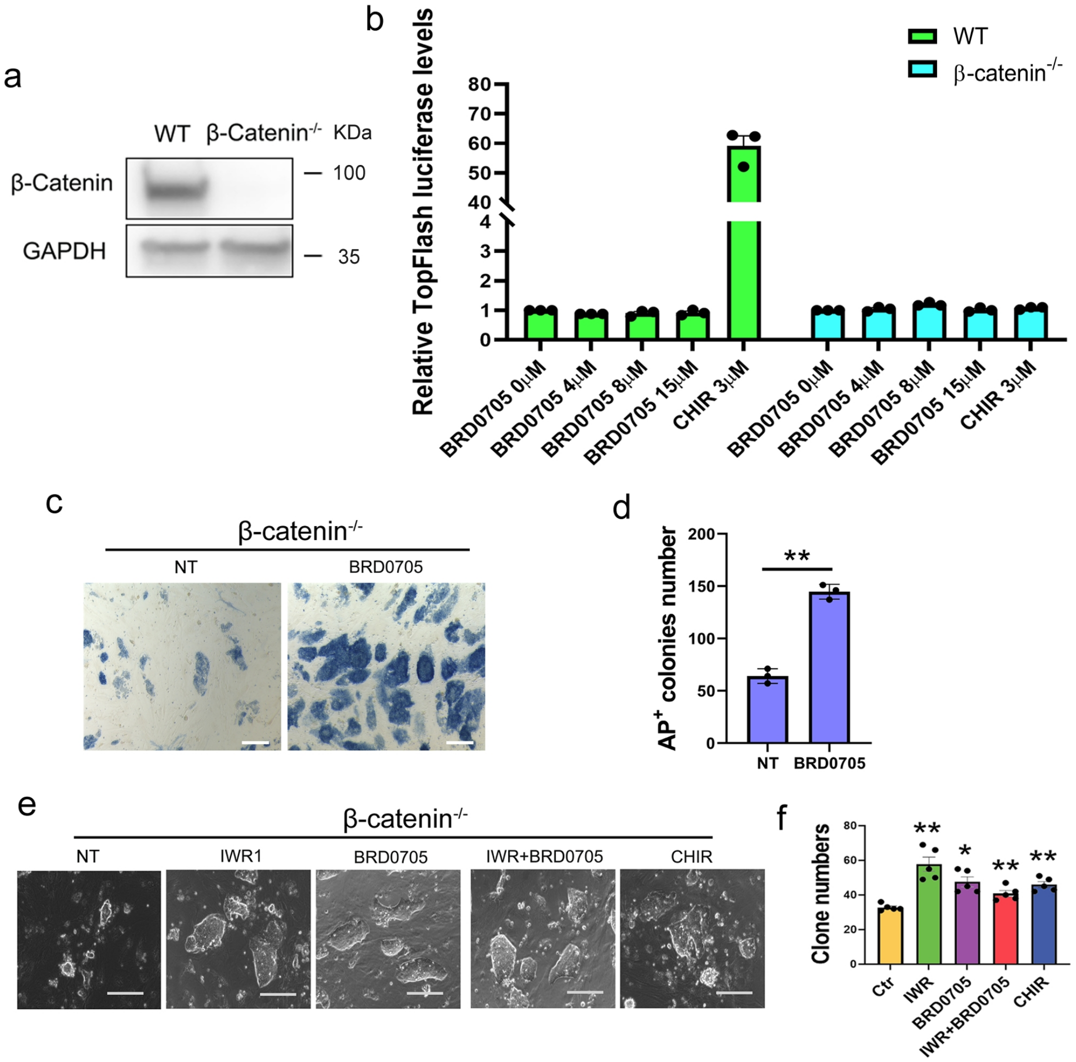

Supplementary information, Fig. S7. BRD0705 promotes ESC self-renewal in β-Catenin$^{-/-}$ ESCs without activating canonical Wnt/β-Catenin signaling.

a Western blot analysis confirming the absence of β-catenin protein in β-Catenin$^{-/-}$ ESCs compared with WT controls. GAPDH served as a loading control.

b Relative TopFlash luciferase reporter activity in WT and β-catenin$^{-/-}$ ESCs treated with increasing concentrations of BRD0705 or CHIR (3 μM). CHIR robustly activated WNT signaling in WT but not in β-catenin$^{-/-}$ ESCs, whereas BRD0705 did not induce WNT pathway activation in either genotype. Data are presented as mean ± SEM.

c Representative AP staining of β-catenin$^{-/-}$ ESCs following treatment with BRD0705 or no treatment (NT). Scale bar: 50 μm.

d Quantification of AP-positive staining in c. Data are presented as mean ± SEM, **, $p < 0.01$.

e Morphological analysis of β-catenin$^{-/-}$ ESCs cultured in LIF/N2B27 on feeders under different treatment conditions. Representative images display cells cultured in NT, IWR1, BRD0705, IWR+BRD0705, or CHIR conditions. Scale bars, 100 μm.

f Quantification of colony numbers corresponding to panel e, showing β-catenin$^{-/-}$ ESCs under the indicated treatments: Ctr, IWR1, BRD0705, BI, and CHIR. Data are presented as mean ± SEM, *, $p < 0.05$, **, $p < 0.01$.

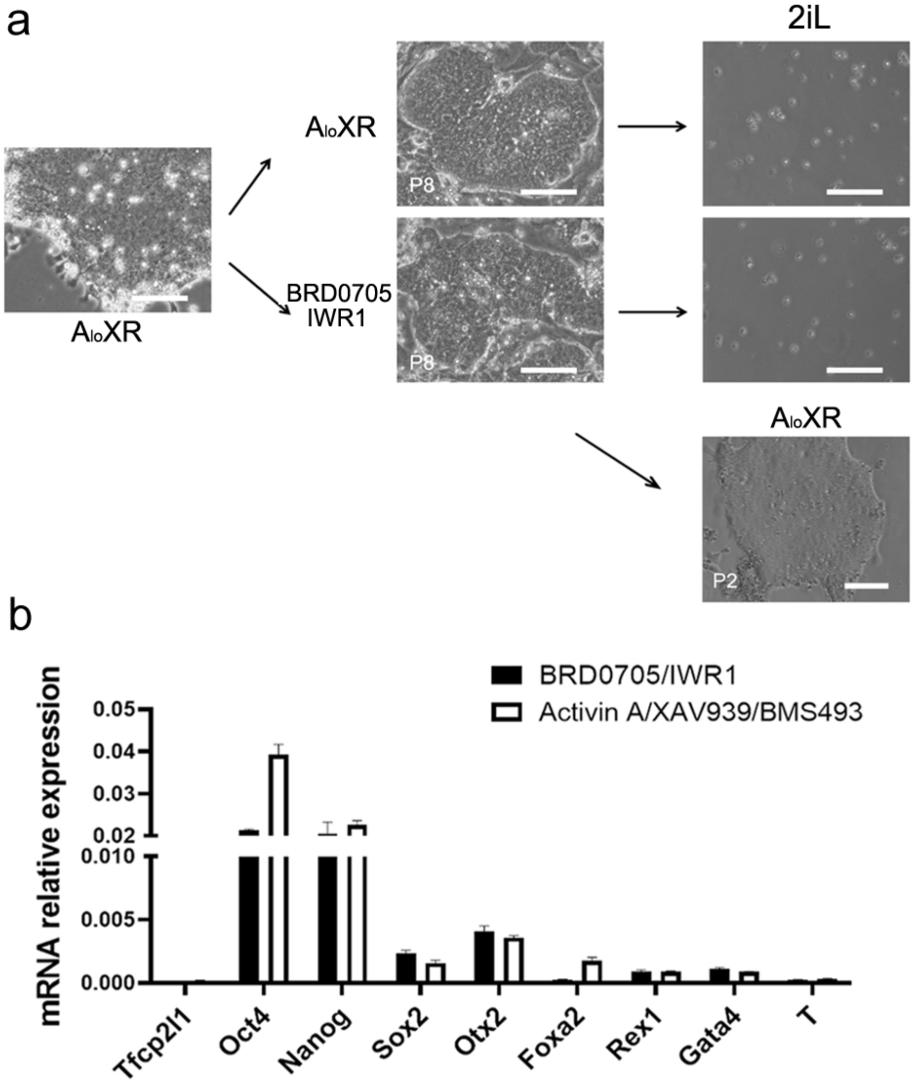

Supplementary information, Fig. S8. BRD0705 effectively maintains the stemness of mouse formative cells.

a Representative images showing the morphology of formative cells cultured under BRD0705/IWR-1 conditions or maintained in $A_{lo}XR$ conditions for eight passages. They were then transitioned to 2iL conditions to assess cell viability, while cells cultured under BI were also reverted to $A_{lo}XR$ as a control. Scale bars, 100 μm.

b qRT-PCR analysis of marker gene expression was performed on formative cells cultured under both BRD0705/IWR-1 and $A_{lo}XR$ conditions, with expression levels normalized to GAPDH. Error bars represent the SEM from technical triplicates.

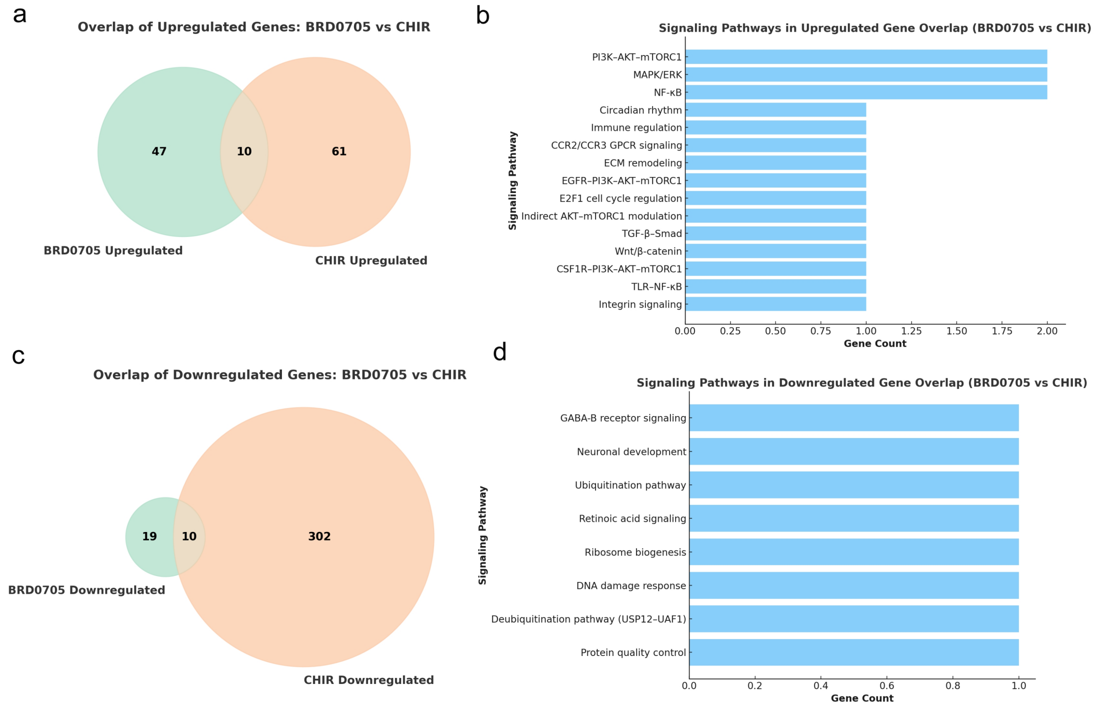

Supplementary information, Fig. S9. RNA-seq analysis of β-catenin$^{-/-}$ ESCs identifies genes upregulated or downregulated by BRD0705 and CHIR. identifies genes.

a Venn diagram showing the overlap of upregulated genes between BRD0705- and CHIR-treated ESCs.

b Enriched signaling pathways associated with the overlapping upregulated genes in **a**, with gene counts indicated on the x-axis.

c Venn diagram showing the overlap of downregulated genes between BRD0705- and CHIR-treated ESCs.

d Enriched signaling pathways associated with the overlapping downregulated genes in **c**, with gene counts indicated on the x-axis.

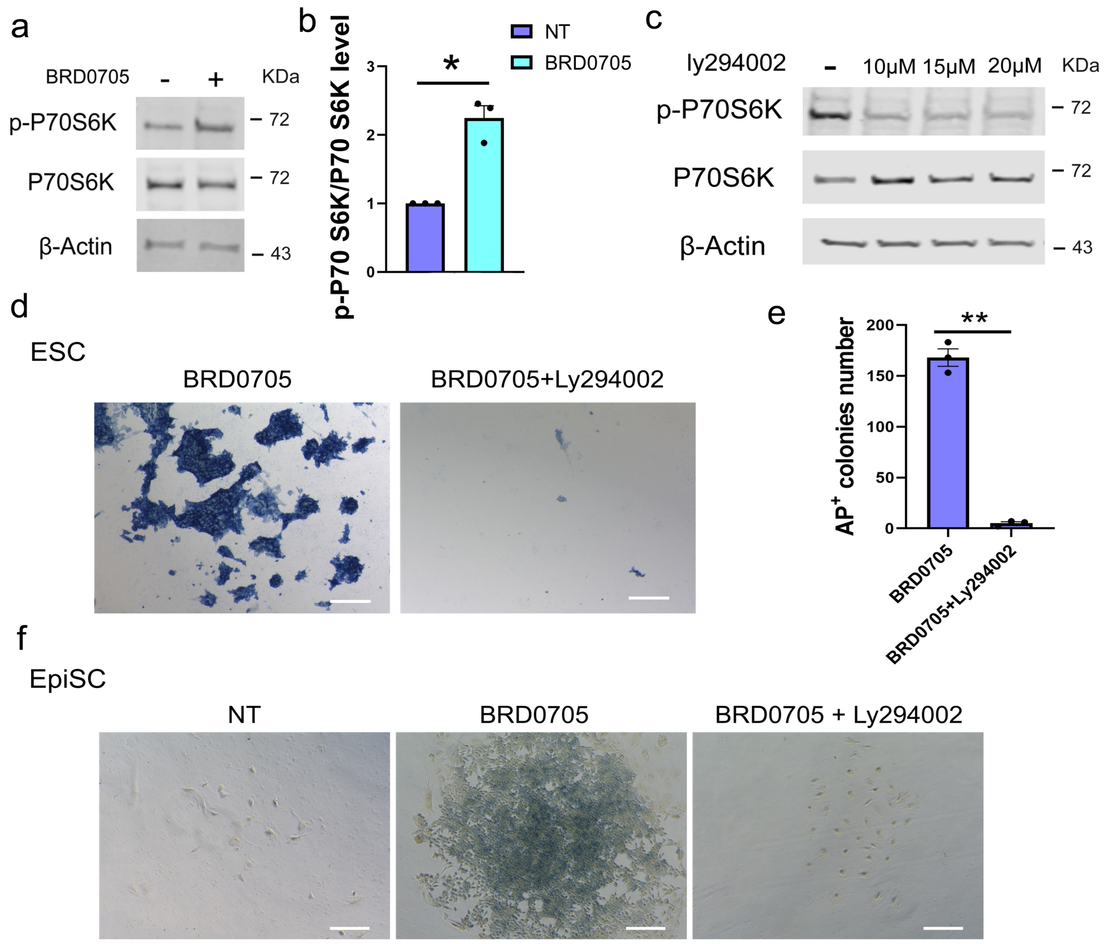

Supplementary information, Figure S10. BRD0705 activates P70S6K signaling and promotes ESC self-renewal in a PI3K-dependent manner.

a Western blot analysis of phosphorylated p70S6K (p-p70S6K), total p70S6K, and β-actin in ESCs treated with or without BRD0705.

b Quantification of the p-p70S6K/p70S6K ratio shown in a. Data represent mean ± SEM; *p* < 0.05.

c Western blot analysis showing dose-dependent suppression of BRD0705-induced p70S6K phosphorylation by the PI3K inhibitor LY294002.

d Representative images of AP staining of ESCs cultured in DMEM/FBS medium treated with BRD0705 alone or BRD0705 plus LY294002 for two passages. Scale bars, 200 μm.

e Quantification of AP^+ colony numbers in d. Data represent mean ± SEM; $p < 0.01$.

f Representative images of AP staining of EpiSCs cultured under NT, BRD0705, or BRD0705 + LY294002 conditions for two passages. Scale bars, 200 μm.

	Small-molecule cocktail	Feeder	ESC self-renewal
DMEM/FBS	BRD0705/IWR1 or BRD0705	No	√
		Yes	√
N2B27		No	×
		Yes	√

Supplementary information, Figure S11. Culture conditions under which BI or BRD0705 supports ESC self-renewal across different media and feeder dependencies.

Supplementary information, Table S1. List of small-molecule library compounds.

Supplementary information, Table S2. Analysis of bulk RNA-seq results from β-catenin$^{-/-}$ ESCs treated with BRD0705, CHIR, and control.

Supplementary information, Table S3. Gene lists of overlapping bivalent genes identified in ESCs and EpiSCs co-cultured under BI conditions and those reported under conventional culture conditions.

Supplementary information, Table S4. List of qPCR primers.

	FW	RV
Tfcp2l1	GGGGACTACTCGGAGCATCT	TGTTTCCGATCAGCTCCCTT
Rex1	AAACGGCAAAGACAAGTGGC	TAGGGTCAGTCTGTCGAGGG
Oct4	GTGGACCTCAGGTTGGACTG	GGAGGTTCCCTCTGAGTTGC
Nanog	TGGAAGCCTTTCCATGTGGG	TATGGAGCGGAGCAGCATTC
Sox2	TTTGTCCGAGACCGAGAAGC	CTCCGGGAAGCGTGTACTTA
Otx2	CCCTGGGCTTCTTGTCCTG	CACCCTGGATTCTGGCAAGT
Foxa2	TGAAGATGGAAGGGCACGAG	CTCACGGAAGAGTAGCCCTC
Gata4	AGCAGGACTCTTGGAACAGC	TACGCGGTGATTATGTCCCC
T	CTACATCCACCCAGACTCGC	CCCCCTCCATTGAGCTTGTT
Gapdh	TTTGCAGTGGCAAAGTGGAGATT	CCCATTTGATGTTAGTGGGGTCTCG
Fgf5	AAAACCTGGTGCACCCTAGA	CATCACATTCCCGAATTAAGC
Foxa2	TGAAGATGGAAGGGCACGAG	CTCACGGAAGAGTAGCCCTC

Supplementary information, Table S5. Key resources table

REAGENT or RESOURCE	SOURCE	IDENTIFIER
Antibodies		
Rabbit polyclonal anti-phospho-β-Catenin (Ser33/37/Thr41)	Cell Signaling Technology	CAT#9561; RRID: AB_331729
Rabbit monoclonal anti-β-catenin	Cell Signaling Technology	CAT#8480; RRID: AB_11127855
Mouse monoclonal anti-GAPDH	Proteintech	CAT#600004-1-Ig; RRID: AB_2107436
Goat polyclonal secondary anti-rabbit IgG, HRP-linked Antibody	Cell Signaling Technology	CAT#7074; RRID: AB_2099233
Horse polyclonal secondary anti-mouse IgG, HRP-linked Antibody	Cell Signaling Technology	CAT#7076; RRID: AB_330924
Rabbit monoclonal anti-GSK3α/β	Cell Signaling Technology	CAT#5676; RRID: AB_10547140
Rabbit monoclonal anti-OCT4	Thermo Fisher Scientific	CAT#MA5-14845; RRID: AB_10979606
Rabbit monoclonal anti-NANOG	Thermo Fisher Scientific	CAT#500-P236-50ug; RRID: AB_2929968
Donkey polyclonalanti-Rabbit IgG (H+L), Alexa Fluor™ 555	Invitrogen	CAT#A31572; RRID: AB_162543
Rabbit monoclonal anti-TUJ1	Cell Signaling Technology	CAT#5568; RRID: AB_10694505
Rabbit monoclonal anti-FOXA2	Cell Signaling Technology	CAT#8186; RRID: AB_10891055
Mouse monoclonal anti-MF 20	Developmental Studies Hybridoma Bank	CAT#MF 20; RRID: AB_2147781
Rabbit polyclonal anti-H3K4me3	Active Motif	CAT#39060; RRID: AB_2615077
Mouse monoclonal anti-H3K27ac	Active Motif	CAT#39685; RRID: AB_2793305
Rabbit polyclonal anti-H3K27me3	Active Motif	CAT# 39055; RRID: AB_2561020

Chemicals, peptides, and recombinant proteins		
BRD0705	Cayman	CAT#37314
IWR1	Selleckchem	CAT#S7086
Recombinant Human LIF	Gibco	CAT#300-05-5UG
CHIR-99021	Selleckchem	CAT#S1263
PD0325901	Selleckchem	CAT#S1036
Recombinant Human FGF2	Gibco	CAT#100-18B-100UG
Recombinant Human/Mouse/Rat Activin A	Gibco	CAT#120-14P-10UG
XAV-939	Selleckchem	CAT#S1180
BMS493	Selleckchem	CAT#E1627
3MB-PP1	Cayman Chemical	CAT#17860
N2 Supplement	Gibco	CAT#17502048
B27 Supplement	Gibco	CAT#17504044
Neurobasal medium	Gibco	CAT#21103049
DMEM/F12 medium	Gibco	CAT#11320033
Gelatin	Sigma-Aldrich	CAT#G1890-500G
L-Glutamin	Gibco	CAT#25030081
MEM NEAA	Gibco	CAT#11140050
Sodium Pyruvate	Gibco	CAT#11360070
IMDM	Gibco	CAT#12440053
2-Mercaptoethanol	Sigma-Aldrich	CAT#M3148
DMEM	Gibco	CAT#11965092
Critical commercial assays		
Alkaline Phosphatase Kit	Vector laboratories	CAT#SK-5300
RNeasy Mini Kit	Qiagen	CAT#74104
iTaq™ Universal SYBR® Green Supermix	Bio-Rad	CAT#1725124

CUTANA CUT&Tag Kit	Epicypher	CAT#15-1017t
Chromium Next GEM Single Cell 3' Kit v3.1	10x Genomics	CAT# PN-1000268
Deposited data		
CUT&TAG	This paper	GEO: GSE295265; https://www.ncbi.nlm.nih.gov/geo/query/acc.cgi?acc=GSE295265
scRNA-seq	This paper	GEO: GSE295264; https://www.ncbi.nlm.nih.gov/geo/query/acc.cgi?acc=GSE295264
Bulk RNA-seq	This paper	GEO: GSE295263; https://www.ncbi.nlm.nih.gov/geo/query/acc.cgi?acc=GSE295263
Bulk RNA-seq	This paper	GEO: GSE304689; https://www.ncbi.nlm.nih.gov/geo/query/acc.cgi?acc=GSE304689
Experimental models: Cell lines		
Mouse ES-E14 cell line	Ying et al.[12]	RRID: CVCL_C320
Mouse ES-46C cell line	Ying et al.[7]	RRID: CVCL_Y482
Mouse Oct4-GiP ES cell line	Ying et al.[2]	N/A
Mouse CD1-EpiSCs	Kim et al.[3]	N/A
Mouse B6D2F1 ESCs-2i	This paper	N/A
Mouse B6D2F1 ESCs-BRD0705/IWR1	This paper	N/A
GFP-puromycin mouse ESCs	This paper	N/A
GFP-puromycin mouse CD1-EpiSCs	This paper	N/A
RFP-zeocin mouse ESCs	This paper	N/A
Gsk3α$^{-/-}$; Gsk3β$^{-/-}$ mouse ES cells	Chen et al.[8]	N/A
Gsk3α$^{-/-}$ mouse ES cells	Chen et al.[8]	N/A

Gsk3β$^{-/-}$ mouse ES cells	Chen et al.[8]	N/A
Gsk3α$^{-/-}$+Gsk3α (L195G) mouse ES cells	Chen et al.[8]	N/A
Gsk3β$^{-/-}$+Gsk3β (L132G) mouse ES cells	Chen et al.[8]	N/A
Gsk3α$^{-/-}$+Gsk3α (K148R) mouse ES cells	Chen et al.[8]	N/A
β-catenin$^{-/-}$ mouse ES cells	Kim et al.[3]	N/A
Experimental models: Organisms/strains		
Mouse/B6D2F1	Charles river	099
Mouse/C57BL/6J	Charles river	027
Oligonucleotides		
primers for qRT-PCR	See Table S3	N/A
Recombinant DNA		
PiggyBac vector (CAG promoter)	Ye et al.[13]	N/A
Software and algorithms		
Fiji-ImageJ	National Institutes of Health	https://imagej.net/Fiji
GraphPad Prism 9	GraphPad	https://www.graphpad.com/scientific-software/prism/
Snapgene	Snapgene	https://www.snapgene.com
Python 3.7.13	Python	https://www.python.org/
R 4.1.3	R package	https://cran.r-project.org/mirrors.html
MACS2 v2.2.9.1	Zhang et al.[14]	https://github.com/taoliu/MACS
Bowtie version 1.2.1.1	Langmead et al.[15]	http://bowtie-bio.sourceforge.net/index.shtml
BEDTools	Quinlan et al.[16]	https://github.com/arq5x/bedtools2; RRID: SCR_006646

STAR	Dobin et al.[17]	https://code.google.com/archive/p/rna-star/; RRID: SCR_004463
DESeq2 v1.40.2	Love et al.[18]	http://www.bioconductor.org/packages/release/bioc/html/DESeq2.html.
Seurat	Satija et al.[19]	https://satijalab.org/seurat/articles/get_started.html; RRID: SCR_016341
CellRanger toolkit (version 6.1.2)	10x Genomics	http://10xgenomics.com/
FlowJo 10.7.1	FlowJo	https://www.flowjo.com/solutions/flowjo; RRID: SCR_008520

Supplementary information, Video S1. Beating of GFP^+ muscle cells in E10.5 mouse embryos obtained by injecting wild-type blastocysts with GFP-labeled ESCs cultured with BRD0705/IWR1.

References

1 Kinoshita, M. *et al.* Capture of Mouse and Human Stem Cells with Features of Formative Pluripotency. *Cell stem cell* **28**, 453-471 e458 (2021).

2 Ying, Q. L., Nichols, J., Evans, E. P. & Smith, A. G. Changing potency by spontaneous fusion. *Nature* **416**, 545-548 (2002).

3 Kim, H. *et al.* Modulation of β-catenin function maintains mouse epiblast stem cell and human embryonic stem cell self-renewal. *Nature communications* **4**, 2403 (2013).

4 Zhou, X., Chadarevian, J. P., Ruiz, B. & Ying, Q. L. Cytoplasmic and Nuclear TAZ Exert Distinct Functions in Regulating Primed Pluripotency. *Stem cell reports* **9**, 732-741 (2017).

5 Huang, X. & Wu, S. M. Isolation and functional characterization of pluripotent stem cell-derived cardiac progenitor cells. *Current protocols in stem cell biology* **Chapter 1**, Unit 1F.10 (2010).

6 Ying, Q. L. & Smith, A. G. Defined conditions for neural commitment and differentiation. *Methods in enzymology* **365**, 327-341 (2003).

7 Ying, Q. L., Stavridis, M., Griffiths, D., Li, M. & Smith, A. Conversion of embryonic stem cells into neuroectodermal precursors in adherent monoculture. *Nature biotechnology* **21**, 183-186 (2003).

8 Chen, X. *et al.* A Chemical-Genetic Approach Reveals the Distinct Roles of GSK3α and GSK3β in Regulating Embryonic Stem Cell Fate. *Developmental cell* **43**, 563-576.e564 (2017).

9 Deng, Q., Ramsköld, D., Reinius, B. & Sandberg, R. Single-cell RNA-seq reveals

dynamic, random monoallelic gene expression in mammalian cells. *Science (New York, N.Y.)* **343**, 193-196 (2014).

10 Mohammed, H. *et al.* Single-Cell Landscape of Transcriptional Heterogeneity and Cell Fate Decisions during Mouse Early Gastrulation. *Cell reports* **20**, 1215-1228 (2017).

11 Chen, G. *et al.* Single-cell analyses of X Chromosome inactivation dynamics and pluripotency during differentiation. *Genome research* **26**, 1342-1354 (2016).

12 Ying, Q. L. *et al.* The ground state of embryonic stem cell self-renewal. *Nature* **453**, 519-523 (2008).

13 Ye, S. *et al.* Wnt/β-catenin and LIF-Stat3 signaling pathways converge on Sp5 to promote mouse embryonic stem cell self-renewal. *Journal of cell science* **129**, 269-276 (2016).

14 Zhang, Y. *et al.* Model-based analysis of ChIP-Seq (MACS). *Genome biology* **9**, R137 (2008).

15 Langmead, B., Trapnell, C., Pop, M. & Salzberg, S. L. Ultrafast and memory-efficient alignment of short DNA sequences to the human genome. *Genome biology* **10**, R25 (2009).

16 Quinlan, A. R. & Hall, I. M. BEDTools: a flexible suite of utilities for comparing genomic features. *Bioinformatics (Oxford, England)* **26**, 841-842 (2010).

17 Dobin, A. *et al.* STAR: ultrafast universal RNA-seq aligner. *Bioinformatics (Oxford, England)* **29**, 15-21 (2013).

18 Love, M. I., Huber, W. & Anders, S. Moderated estimation of fold change and dispersion for RNA-seq data with DESeq2. *Genome biology* **15**, 550 (2014).

19 Satija, R., Farrell, J. A., Gennert, D., Schier, A. F. & Regev, A. Spatial reconstruction of single-cell gene expression data. *Nature biotechnology* **33**, 495-502 (2015).
